# The B-type response regulator GmRR11d mediates systemic inhibition of symbiotic nodulation

**DOI:** 10.1038/s41467-022-35360-9

**Published:** 2022-12-10

**Authors:** Jiahuan Chen, Zhijuan Wang, Lixiang Wang, Yangyang Hu, Qiqi Yan, Jingjing Lu, Ziyin Ren, Yujie Hong, Hongtao Ji, Hui Wang, Xinying Wu, Yanru Lin, Chao Su, Thomas Ott, Xia Li

**Affiliations:** 1 National Key Laboratory of Crop Genetic Improvement, Hubei Hongshan Laboratory, College of Plant Science and Technology, Huazhong Agricultural University, Wuhan, China; 2 College of Agriculture, Shanxi Agricultural University, Taigu, China; 3 University of Freiburg, Faculty of Biology, Cell Biology, Freiburg, Germany; 4 CIBSS - Centre of Integrative Biological Signalling Studies, University of Freiburg, Freiburg, Germany; 5 Guangdong Laboratory for Lingnan Modern Agriculture, Wushan Road, Guangzhou, Guangdong, PR China

**Keywords:** Plant signalling, Rhizobial symbiosis, Plant hormones

## Abstract

Key to the success of legumes is the ability to form and maintain optimal symbiotic nodules that enable them to balance the trade-off between symbiosis and plant growth. Cytokinin is essential for homeostatic regulation of nodulation, but the mechanism remains incompletely understood. Here, we show that a B-type response regulator GmRR11d mediates systemic inhibition of nodulation. *GmRR11d* is induced by rhizobia and low level cytokinin, and GmRR11d can suppress the transcriptional activity of GmNSP1 on *GmNIN1a* to inhibit soybean nodulation. GmRR11d positively regulates cytokinin response and its binding on the *GmNIN1a* promoter is enhanced by cytokinin. Intriguingly, rhizobial induction of *GmRR11d* and its function are dependent upon GmNARK that is a CLV1-like receptor kinase and inhibits nodule number in shoots. Thus, GmRR11d governs a transcriptional program associated with nodulation attenuation and cytokinin response activation essential for systemic regulation of nodulation.

## Abstract (web summary)

Cytokinin is essential for regulation of nodulation. Here, the authors identified a B-type response regulator GmRR11d that governs a transcriptional program associated with nodulation and cytokinin activation essential for systemic regulation of nodulation.

## Introduction

Symbiotic nodulation is imperative for the survival and productivity of legumes under nitrogen-limiting conditions. It is initiated upon plant perception of lipochitooligosaccharide Nod factors (NFs) released by rhizobia^1,2^ and requires the coordination of rhizobial infection and root nodule organogenesis. The LysM-type receptor kinases (e.g., NFR1 and NFR5 in *Lotus* (*L*.) *japonicus*, NFP in *Medicago* (*M*.) *truncatula* and GmNFR1α and GmNFR5α in soybean)^3–9^ recognize NFs and activate the NF signaling pathway involving the encoding/decoding of nuclear Ca^2+^oscillations, heteropolymerization of NODULATION SIGNALING PATHWAY 1 (NSP1)-NSP2 and activation of nodulation genes, such as *NODULE INCEPTION* (*NIN*)^10–12^.

Nodule initiation largely depends on the locally produced phytohormone cytokinin (CKs) to allow root cortical cells to successfully dedifferentiate and proliferate in the initial stages of nodulation^13–17^. The levels of CKs in plants are regulated by the biosynthesis and metabolism of CKs. Here, the most prevalent active CK is *trans*-zeatin (*tZ*) and its biosynthesis is regulated by Isopentenyl transferases (IPTs) and the cytochrome P450 enzymes CYP735A1/2^18,19^. The free base in active forms of CKs is catalyzed by LONGLY GUY (LOG) family enzymes CK nucleoside 5′-monophosphate phosphoribohydrolases^20,21^. CK levels are also modulated by sugar conjugation or via irreversible cleavage by cytokinin oxidases (CKXs)^22^. The biosynthesis and metabolism of CKs are dynamically regulated by endogenous developmental cues as well as abiotic and biotic stimuli^18,22^. In legumes, the expression of multiple CK synthesis genes is rapidly induced by rhizobia and NFs, resulting in an early burst of CKs^23,24^. Mutations in CK synthesis genes, such as *ISOPENTENYL TRANSFRASE 3* (*IPT3*) and *IPT4* as well as several *LOG* genes (*LOG1/2/4*) result in reduced nodulation^25–28^. Analogously, loss of function of the *CYTOKININ OXIDASE/DEHYDROGENASE 3* (*LjCKX3*) gene, which causes elevated root CK levels, also reduces the number of nodules^24^. Thus, the CK bioactive pool is precisely regulated during nodulation to maintain an optimal number of nodules in the root system.

Plants perceive CKs via CK sensors histidine kinases (HKs) and transmit the signals to type-B response regulators (RRs) through authentic histidine phosphotransferase (AHPs), which then propagate the signaling by activating primary CK-responsive genes, including type-A RR genes^29^. The type-B RRs contain a receiver domain and a C-terminal extension including a Myb-like DNA binding domain. These type-B genes are not transcriptionally regulated by CKs, but they can directly bind to the specific motifs in the promoters of many CK-responsive genes in a CK-dependent manner to activate or repress these genes^29,30^. Thus, the type-B RRs are essential for the initial response to CKs at transcriptional level and can interact with transcription factors, such as TGA3 in salicylic acid (SA) and DELLA proteins in gibberellin (GA), to mediate the cross talks between CK and these hormonal signaling pathways^31,32^. Rhizobia inoculation can induce CK sensor genes, such as *MEDICAGO CYTOKININ RESPONSE 1* (*MtCRE1*) and *LOTUS HISTIDINE KINASE 1* (*LHK1*), to activate the CK signaling that is essential for nodulation^23,29,33–35^. Genetic mutations that impair CK perception and response can lead to altered nodulation. For example, gain-of-function mutations in *MtCRE1* and *LjLHK1* induce spontaneous nodules in the absence of rhizobia^33,35^, while loss-of-function mutations in *MtRRB3* lead to the reduction of the nodulation^36^. Genetic data suggest that CKs act downstream of the CALCIUM-AND CALMODULIN-DEPENDENT PROTEIN KINASE (CCaMK) but upstream of NIN in the NF signaling pathway^33^. Notably, CKs induce the expression of *NIN*^16^, which conversely activates *MtCRE1*^37^. CK-induced *MtNIN* also activates the symbiosis-specific gene *MEDICAGO C-TERMINALLY ENCODED PEPTIDE 7* (*MtCEP7*) to systemically positively regulate nodulation through its receptor, LRR-RLK COMPACT ROOT ARCHITECTURE 2 (MtCRA2), in shoots^38^. Therefore, CK and NF signaling pathways may converge at NIN to control root cellular fate and subsequent nodule formation.

Symbiotic nodulation is achieved at the expense of legume growth. Plants have evolved an intrinsic control mechanism, called autoregulation of nodulation (AON), to systemically regulate nodulation so that plants can balance the carbon costs of growth and nodulation. During nodule formation, CK-induced NINs can directly activate the CLAVATA3/EMBRYO (CLE) coding genes *MtCLE12/13*, lotus *CLE-ROOT SIGNAL 1/2/3* (*LjCLE-RS1/2/3*), and soybean *RHIZOBIA-INDUCED CLE 1/2* (*GmRIC1/2*)^39–41^. These peptide hormones are transported to the shoot to bind to their receptors, such as SUPER NUMERARY NODULE (SUNN), HYPERNODULATION AND ABERRANT ROOT 1 (HAR1), and *Glycine max* NODULE AUTOREGULATION RECEPTOR KINASE (GmNARK), which negatively regulate nodulation using long distance signals^42–45^. The CKs synthesized in shoots have been proposed to function as mobile signals to inhibit nodulation by suppressing *NIN*^26,46^. It has long been proposed that shoot-controlled nodulation acts downstream of CK signaling-mediated activation of nodule initiation^33^. Additionally, Lotus uses an LHK1-dependent feedback mechanism from the root cortex to the epidermis, which is coupled to AON to monitor and maintain rhizobial infection and nodule development^47^. Thus, CKs may trigger the onset of both local and systemic negative feedback loops through NIN. Given the important role of CKs in nodulation homeostasis, there has been increased interest in CK-mediated local and systemic regulation of nodulation. However, the mechanisms that modulate CKs to elicit an optimal response of legumes to rhizobia are not fully understood.

In this work, we identified a B-type RR transcription factor GmRR11d as an important effector of AON that represses *GmNIN1a* expression. We showed that *GmRR11d* expression is responsive to both CKs and rhizobia, but oppositely regulates plant response to CKs and nodulation in soybean. GmRR11d can directly bind to the promoter of *GmNIN1a* to repress its transcription, and CKs can enhance the binding of GmRR11d to *GmNIN1a* promoter. In addition, we found that GmRR11d interacts with GmNSP1a, which is an upstream transcriptional activator of *GmNIN1a*, to suppress its transcriptional activity on *GmNIN1a* expression. We also demonstrated that GmRR11d functions downstream of GmNARK to control root nodulation. Collectively, our findings elucidate a molecular mechanism that regulates nodulation homeostasis by elevating the CK sensitivity of infected root and by attenuating CK-activated nodulation via a systemic negative feedback loop.

## Results

### Identification of GmRR11d as a GmNSP1-interacting protein

As NSP1 activates the expression of *NIN*, which is an evolutionarily conserved and coordinating regulator of CK-mediated nodulation and nodulation autoregulation in legumes^46,48^, we hypothesized that AON might induce a transcription factor that suppresses the transcriptional activity of NSP1, resulting in repression of *NIN* and attenuation of nodulation. To test this hypothesis, we first examined the expression of two soybean *NSP1* ortholog genes (*GmNSP1a* and *GmNSP1b*) and evaluated the effect of individual loss-of-function mutations in *GmNSP1a* and *GmNSP1b* on nodulation of soybean. *GmNSP1a* and *GmNSP1b* were differentially expressed during rhizobial infection and nodule development. *GmNSP1a* was induced by rhizobia rapidly and remained at higher expression levels during nodulation. *GmNSP1b* was initially downregulated after rhizobial inoculation; it was then upregulated during rhizobial early infection and peaks when nodule formed, and then gradually decreasing during nodule development (Supplementary Fig. 1a, b). Gene knockout using CRISPR/Cas9 and phenotypic analysis showed that loss of function mutations in *GmNSP1a* and *GmNSP1b* resulted in reduced numbers of nodules, suggesting that both *GmNSP1a* and *GmNSP1b* positively regulate nodulation in soybean (Supplementary Fig. 1c–g; Supplementary Fig. 2).

Next, we performed a yeast-two-hybrid (Y2H) screen in a soybean nodulation cDNA library using GmNSP1a as a bait to identify the GmNSP1a-interacting proteins. Interestingly, a RR protein (Glyma.18G010800) was identified as a putative GmNSP1a-interacting protein. This protein is the soybean homolog of *Arabidopsis* RR11 (ARR11) (Supplementary Fig. 3), a B-type RR protein^49^. Genome-wide analyses identified four copies of *ARR11* genes (*Glyma.11G246400*, *Glyma.05G144500*, *Glyma.08G100900* and *Glyma.18G010800*) in soybean, which were named *GmRR11a, GmRR11b, GmRR11c* and *GmRR11d* based on their protein homology to ARR11 (Supplementary Fig. 3). These GmRR11s contain conserved receiver domains (REC) and MYB (DNA-binding domain) domains, similar to their putative orthologs in *M. truncatula*, *L. japonicus* and *A. thaliana* (Supplementary Fig. 4a). The putative GmNSP1a-interacting protein GmRR11d is the shortest one compared to GmRR11a/b/c, with significant sequence variations in its C-terminal amino acids compared with ARR11 (Supplementary Fig. 4b).

**Fig. 1:**
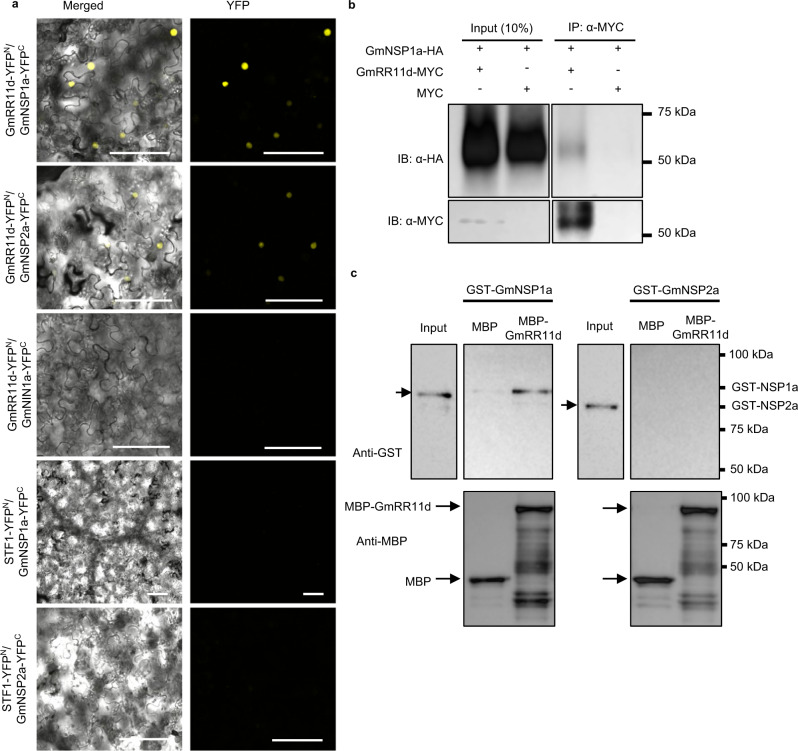
GmRR11d interacts preferentially with GmNSP1a. **a** BiFC assay to detect the interactions between GmRR11d and GmNSP1a or GmNSP2a. The interactions between GmRR11d and GmNIN1a^41^, GmNSP1a or GmNSP2a, and STF1^83^ were used as negative controls. Four independent experiments were repeated with similar results. Scale bars = 100 μm. **b** CoIP assay of interaction between GmRR11d and GmNSP1a. GmRR11d-6xMYC (58 kDa) alone or GmRR11d-6xMYC and GmNSP1a-3xHA (63 kDa) were transiently expressed under the control of the 35 S promoter in *N. benthamiana* leaves. GmRR11d-6xMYC immunoprecipitated from total protein extracts. Three independent experiments were repeated with similar results. **c** Pull-down assays to show that MBP -GmRR11d (91 kDa) interacts with GST-GmNSP1a (87 kDa) but not GST-GmNSP2a (82 kDa). Three independent experiments were repeated with similar results.

To validate the interaction between GmRR11d and GmNSP1a, we performed Y2H, bimolecular fluorescence complementation (BiFC), co-immunoprecipitation (CoIP), pull-down and localized surface plasmon resonance (LSPR) assays. The interaction between GmRR11d and GmNSP1a was detected in both *S. cerevisiae* cells and in the nucleus of *N. benthamiana* leaf cells (Supplementary Fig. 5 and Fig. 1a), which is consistent with the nuclear localization of GmRR11d and GmNSP1a (Supplementary Fig. 6). The in vivo interaction between GmRR11d and GmNSP1a was confirmed by CoIP (Fig. 1b). The in vitro direct interaction between GmRR11d and GmNSP1a was verified by pull-down and LSPR assays (Fig. 1c and Supplementary Fig. 7). NSP1 promotes nodulation by forming a co-transcriptional activator complex with NSP2^48^. There are two soybean *NSP2* ortholog genes (named *GmNSP2a* and *GmNSP2b*) (Supplementary Fig. 8). Both *GmNSP2* genes showed a similar pattern of induction by rhizobia and are required for nodulation of soybean (Supplementary Fig. 9). To test whether the GmRR11d-GmNSP1a interaction is specific, we analyzed whether GmRR11d could interact with GmNSP2 using GmNSP2a as an example. GmNSP2a was present in the nucleus (Supplementary Fig. 6), which is different from the reticulum/nuclear envelope localization of MtNSP2 before NF treatment^12^. It appeared that GmRR11d did not interact with GmNSP2a in either the Y2H, CoIP or the pull-down assays, but it did interact with GmNSP2a in *N. benthamiana* leaf cells and LSPR assay (Fig. 1a, c and Supplementary Fig. 5 and Supplementary Fig. 7). These results suggest that GmRR11d, a B-type RR protein, may be involved in GmNSP1a-regulated nodulation pathway.

### GmRR11d positively regulates root sensitivity to cytokinin

**Fig. 2:**
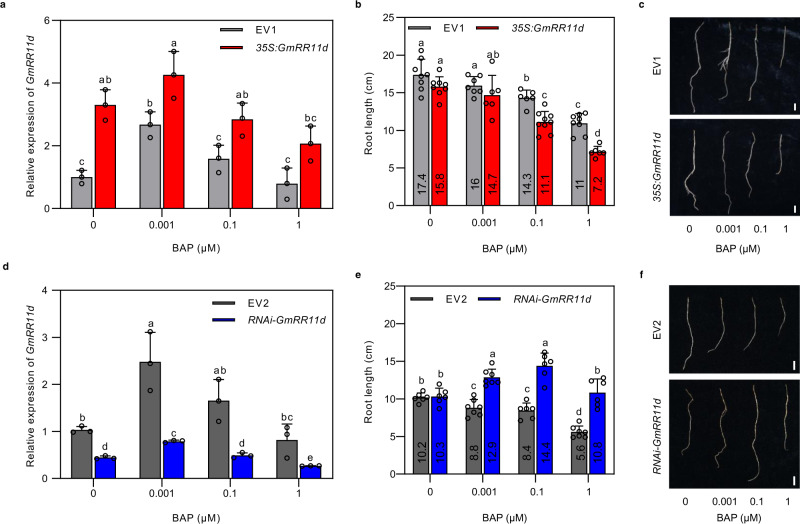
GmRR11d is CK responsive and positively regulates the sensitivity of soybean roots to CKs. **a** Relative expression of *GmRR11d* in transgenic roots expressing *35* *S*:*GmRR11d* treated with BAP of different concentrations (0, 0.001, 0.1, 1 μM). The expression levels were normalized against the geometric mean of soybean *GmELF1b*. Data are presented as means ± SD from three biological replicates. Different letters indicate significant differences at *p* < 0.05 (Two-way ANOVA). **b** Length of hairy roots expressing EV1 and *35* *S*:*GmRR11d* treated with different concentrations (0, 0.001, 0.1, 1 μM) of BAP for 6 days (*n* ≥ 6). Data are presented as means ± SE. Different letters indicate significant differences at *p* < 0.05 (Two-way ANOVA). **c** Representative images of individual root overexpressing *GmRR11d* and the EV1. Three independent experiments were repeated with similar results. Scale bars = 1 cm. **d** Relative expression of *GmRR11d* in transgenic roots expressing *RNAi*-*GmRR11d* treated with BAP of different concentrations (0, 0.001, 0.1, 1 μM). The expression levels were normalized against the geometric mean of soybean *GmELF1b*. Data are presented as means ± SD from three biological replicates. Different letters indicate significant differences at *p* < 0.05 (Two-way ANOVA). **e** Length of hairy roots expressing EV2 and *RNAi*-*GmRR11d* treated with different concentrations (0, 0.001, 0.1, 1 μM) of BAP for 6 days (*n* ≥ 6). Data are presented as means ± SE. Different letters indicate significant differences at *p* < 0.05 (Two-way ANOVA). **f** Representative images of individual root downregulating *GmRR11d* and the EV2. Three independent experiments were repeated with similar results. Scale bars = 1 cm.

ARR11 plays an important role in the CK-induced root growth response in *Arabidopsis*^49^. To explore whether GmRR11d mediates the root response to cytokinin 6-Benzylaminopurine (BAP) in soybean, we expressed *GmRR11d* driven by the CaMV35S (35 S) promoter in the wild-type (Wm82), and the empty vector (EV) was used as a control. qRT-PCR analysis showed that *GmRR11d* expression was significantly induced by low concentration of BAP (0.001 μM) but returned to the basal level in high concentration of BAP in roots expressing empty vector (EV1); the transgenic roots expressing *35* *S:GmRR11d* exhibited markedly elevated expression of *GmRR11d* compared with the vector control roots regardless of BAP concentration (Fig. 2a). Intriguingly, the transgenic roots overexpressing *GmRR11d* exhibited enhanced sensitivity to cytokinin-induced growth inhibition compared to the vector control (Fig. 2b, c). To validate the role of *GmRR11d* in CK response of soybean, we also analyzed the CK sensitivity of the *RNAi-GmRR11d* hairy roots. As expected, *RNAi-GmRR11d* transgenic roots exhibited substantially reduced cytokinin sensitivity (Fig. 2d–f). These results indicate that *GmRR11d* acts as a positive regulator of CK signaling and the plant CK response in soybean.

To confirm the effect of *GmRR11d* on the plant response to cytokinin, we analyzed the expression levels of *GmRR15a* and *GmRR15b*, the closest homologs of *Arabidopsis ARR7* and *ARR15* (Supplementary Fig. 10), which are CK-inducible type-A *ARR* genes in *Arabidopsis*^50^. Indeed, both *GmRR15a* and *GmRR15b* were induced by exogenous CKs (Supplementary Fig. 11a). In the *GmRR11d*-overexpressing hairy roots, the transcript levels of both genes were significantly higher than that in the vector control roots following cytokinin treatments, especially the level of *GmRR15b* (Supplementary Fig. 11b, c), suggesting that the cytokinin hypersensitive phenotype of *GmRR11d*-overexpressing roots is mainly due to elevated expression of these cytokinin-responsive genes. Together, these results suggest that GmRR11d is a typical type-B transcription factor and regulates the transcriptional and physiological responses of soybean roots to CKs.

### *GmRR11d* is induced by rhizobia and is highly expressed in nodules

**Fig. 3:**
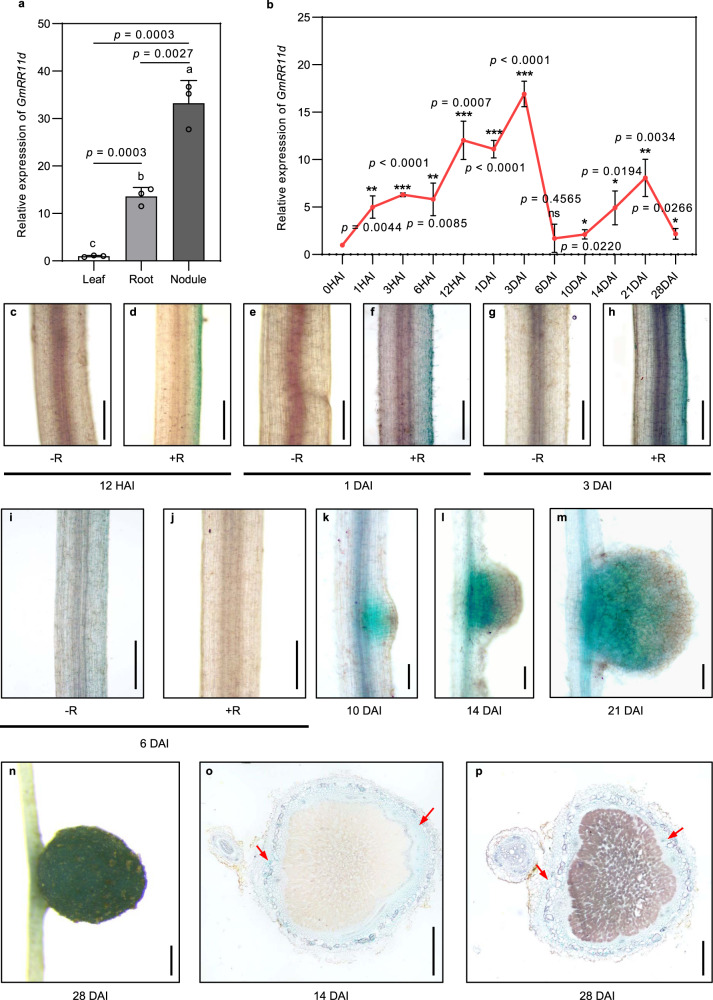
The expression pattern of GmRR11d during nodulation. **a**, **b** qRT-PCR analysis of *GmRR11d* expression in different tissues or during nodulation stages. **a** Roots, leaves, and nodules of soybean seedlings inoculated with *B. diaefficiens* strain USDA110 were harvested at 28 DAI and used for gene expression analyses. *GmELF1b* was used as the endogenous control gene. Data are presented as means ± SD from three biological replicates. More than 6 samples were analyzed in each independent biological repeat. Different letters indicate significant differences at *p* < 0.05 (One-way ANOVA). **b** Seven-day-old seedlings were inoculated with *B. diaefficiens* strain USDA110 and the infected root materials were collected at 0, 1, 3, 6, 12 HAI and 1, 3, 6,10, 14, 21, 28 DAI for expression analysis of *GmRR11d*. *GmELF1b* was used as the endogenous control gene. The data are shown as the means ± SD from three biological replicates. Asterisks indicate significant differences relative to 0 HAI (Two-sided Student’s *t*-test, *p < 0.05; **p < 0.01; ***p < 0.001). **c**–**p** Histochemical assay of *proGmRR11d*:*GUS* and in the transgenic roots and nodules. Three independent experiments were repeated with similar results. **c**–**j** Representative GUS staining in hairy roots of *proGmRR11d*:*GUS*. GUS activity was monitored at 12 HAI **c**, **d**, 1 DAI **e**, **f**, 3 DAI **g**, **h** and 6 DAI **i**, **j** with *B. diazoefficiens* USDA110. Scale bars = 100 μm in **c**–**j**. **k**–**n** The expression pattern of *GmRR11d* in emerging nodule **k**, young nodule **l** and mature nodule **m**, **n**. **o**, **p** Cross-sectional analysis of the expression pattern of *GmRR11d* in young and mature nodules, the red arrows show cortex. Scale bars = 1 mm in **k**–**p**.

To investigate whether *GmRR11d* is involved in nodulation, we first analyzed the expression of *GmRR11d*. Results from qRT-PCR analyses showed that *GmRR11d* was expressed at the highest level in mature nodules compared with in leaves and roots at 28 days after inoculation (DAI) (Fig. 3a). Further gene expression analysis revealed that *GmRR11d* was rapidly induced by rhizobia in soybean roots, peaked at 3 DAI and returned to its original level at 6 DAI (Fig. 3b). *GmRR11d* expression was then gradually increased and reached its second peak when nodule became mature at 21 DAI (Fig. 3b). To further understand the cellular expression pattern of *GmRR11d*, we generated a construct expressing the *GUS* gene under the control of the 2-kb promoter of *GmRR11d*. Histochemical analysis of *GmRR11d* expression showed that *GmRR11d* was induced by rhizobia and expressed throughout the whole nodule formation process (Fig. 3c–p). *GmRR11d* was highly expressed in nodule primordia (Fig. 3k), in basal emerging and young nodules (Fig. 3l, m) and in intact mature nodules (Fig. 3n). Cross-sectional analysis showed that *GmRR11d* was mainly expressed in the cortex of the young and mature nodules (Fig. 3o, p). These results suggest a probable role of *GmRR11d* in nodule organogenesis in soybean.

### GmRR11d functions as a negative regulator in soybean nodulation

**Fig. 4:**
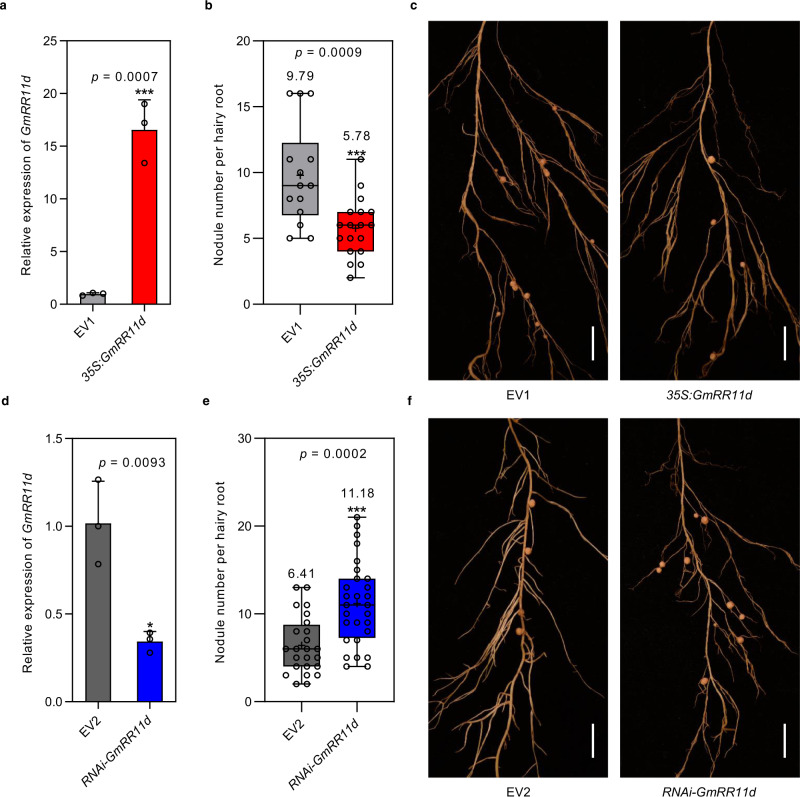
Alterations of *GmRR11d* expression affect nodulation. **a**–**c** Overexpression of *GmRR11d* reduces nodule number. **a** qRT-PCR analysis of transgenic hairy roots expressing *35* *S*:*GmRR11d*. The expression levels were normalized against the geometric mean of soybean *GmELF1b*. Data are presented as means ± SD from three biological replicates. Asterisks indicate significant differences relative to the EV1 control. Two-sided Student’s *t*-test ***p 0.001. **b**, **c** Quantification and representative images of nodule number per hairy root expressing EV1 and *35* *S*:*GmRR11d*. Data are presented as means ± SD (14 hairy roots in EV1 and 18 hairy roots in *35* *S*:*GmRR11d*). Three independent experiments were repeated with similar results. Asterisks indicate significant differences relative to the EV1 control. Two-sided Student’s *t*-test, ***p < 0.001. Scale bars = 1 cm. **d**–**f** Knockdown of *GmRR11d* increases nodule number. **d** qRT-PCR analysis of transgenic hairy roots expressing *RNAi-GmRR11d*. The expression levels were normalized against the geometric mean of soybean *GmELF1b*. Data are presented as means ± SD from three biological replicates. Asterisks indicate significant differences relative to the EV2 control. Two-sided Student’s *t*-test, *p < 0.05. **e**, **f** Nodulation phenotypic analysis of individual hairy roots expressing EV2 and *RNAi-GmRR11d* at 21 DAI. Scale bars = 1 cm. Data are presented as means ± SD (24 hairy roots in EV2 and 28 hairy roots in *RNAi-GmRR11d*). Three independent experiments were repeated with similar results. Asterisks indicate significant differences relative to the EV2 control. Two-sided Student’s *t*-test, ***p < 0.001. Boxes indicate the first and third quartiles and the whiskers indicate the minimum and maximum values, the black lines within the boxes indicate the median values and black circles mark the individual measurements. The numbers above the boxes in (**b**) and (**e**) represent the mean values.

To functionally validate the role of *GmRR11d* in nodulation, we generated hairy roots overexpressing *GmRR11d* (*35* *S*:*GmRR11d*) and evaluated the effect of *35* *S*:*GmRR11d* on nodule number at 21 DAI. Unexpectedly, the *35* *S*:*GmRR11d* roots produced significantly fewer nodules than the control roots by approximately 41% (Fig. 4a–c), suggesting a negative regulatory role of *GmRR11d* in root nodulation of soybean. To investigate whether endogenous *GmRR11d* is a negative regulator of root nodulation, we then generated *RNAi*-*GmRR11d* composite plants to specifically knockdown the expression of *GmRR11d* (Fig. 4d and Supplementary Fig. 12). We found that *GmRR11d* knockdown significantly increased nodule numbers at 21 DAI and the number of root nodules per hairy root was almost doubled (Fig. 4e, f). We also evaluated nodule number of *GmRR11d* knockout roots using CRISPR/Cas9 technology, and the result showed that loss of function mutations in *GmRR11d* caused significantly increased number of nodules (Supplemental Fig. 13a–e). These results confirm that GmRR11d is a key negative regulator of soybean nodulation.

### GmRR11d directly targets and represses the expression of *GmNIN1a*

**Fig. 5:**
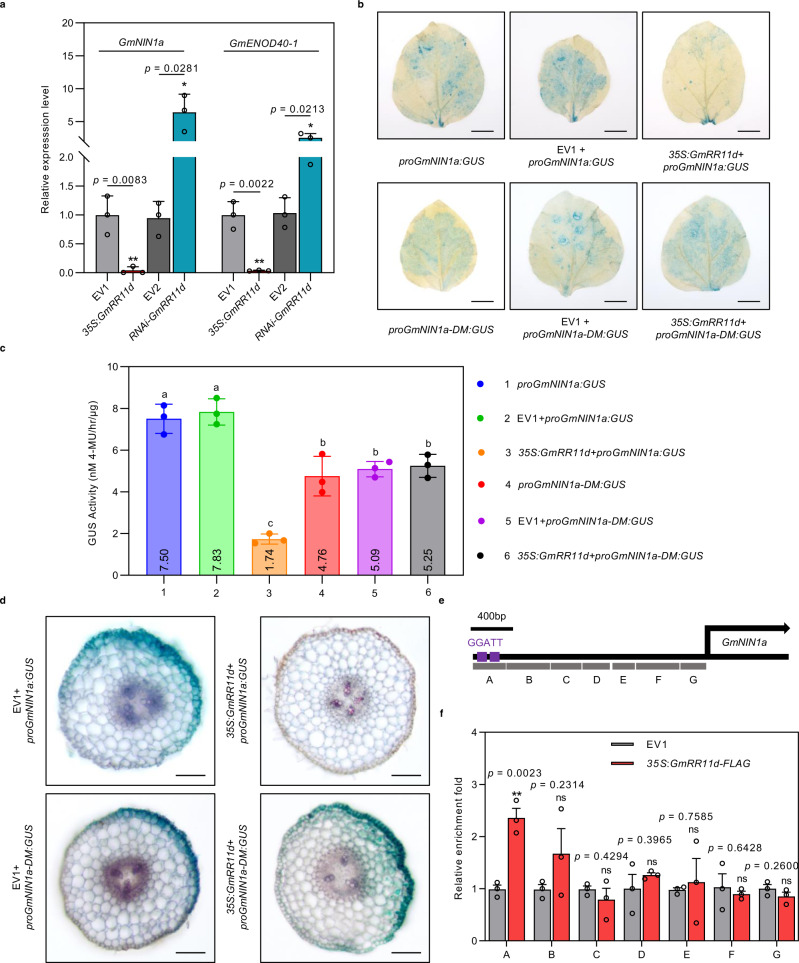
GmRR11d directly targets the promoter of *GmNIN1a* and represses *GmNIN1a* expression. **a** qRT-PCR analysis of *GmNIN1a* and *GmENOD40-1* expression in rhizobia-infected hairy roots expressing *35* *S:GmRR11d* or *RNAi-GmRR11d* at 2 DAI. The expression levels were normalized against the geometric mean of soybean *GmELF1b*. Data are presented as means ± SD from three biological replicates. More than 12 roots were analyzed in three independent biological repeats. Asterisks indicate significant differences relative to the EV1 or EV2 control. Two-sided Student’s *t*-test, *p < 0.05; **p < 0.005. **b**–**d** GUS assays showing the specific binding of GmRR11d to the *GmNIN1a* promoter and the inhibitory effect of GmRR11d on *GmNIN1a* in *N. benthamiana* leaf (**b**, **c**; Scale bars = 1 cm) and in transgenic soybean hairy root (**d**; Scale bars = 100 μm). The *proGmNIN1a* and *proGmNIN1a*-*DM* contain normal (GGATT) and mutated (AAAAA) GmRR11d binding sites in *GmNIN1a* promoter, respectively. The construct harboring *proGmNIN1a:GUS* or *proGmNIN1a-DM* was co-transformed with empty vector (EV1) or *35* *S:GmRR11d* into epidermal cells of *N. benthamiana* leaves and transgenic hairy roots of soybean, and GUS staining were performed 48 h after transformation in *N. benthamiana* leaves and 2 DAI in transgenic hairy roots. Data are presented as means ± SD from three biological replicates. Different letters indicate significant differences at *p* < 0.05 (one-way ANOVA). **e** Diagram of the *GmNIN1a* promoters. GmRR11d binding site (GGATT) shown as purple boxes. The fragments marked by the letters A to G indicate the regions examined in ChIP-qPCR assays. Region A contains two GmRR11d binding sites. **f** ChIP-qPCR assay showing binding of GmRR11d to the *GmNIN1a* promoter. DNA fragments were co-immunoprecipitated with anti-FLAG antibody from chromatin suspensions prepared from *35* *S*:*GmRR11d-FLAG* or empty vector (EV1) samples. DNA fragments corresponding to the regions indicated in **e** were analyzed by qRT-PCR. The DNA fragments were normalized to the input data. Data are presented as mean ± SD of three biological repeats. Asterisks indicate significant differences relative to the EV1 control. Two-sided Student’s *t*-test, **p < 0.01.

The facts that GmRR11d interacts with GmNSP1a and negatively regulates nodulation suggest that GmRR11d may inhibit nodulation by repressing the expression of *GmNIN1a* and its downstream genes. To prove this hypothesis, we examined the expression of *GmNIN1a* and the downstream marker gene *GmENOD40-1* in *35* *S*:*GmRR11d* and *RNAi*-*GmRR11d* hairy roots at 2 DAI. Indeed, the expression of both *GmNIN1a* and *GmENOD40-1* genes was significantly decreased in *35* *S*:*GmRR11d* transgenic roots while increased in *RNAi*-*GmRR11d* transgenic roots (Fig. 5a). To further verify the negative regulatory effect of GmRR11d on *GmNIN1a* transcription, we transiently co-expressed *proGmNIN1a*:*GUS* with empty vector (EV1) or *35* *S*:*GmRR11d* in leaf epidermal cells of *N. benthamiana* leaves. The GUS assay showed that the expression of *GmNIN1a* was markedly decreased when *proGmNIN1a*:*GUS* was co-expressed with *35* *S*:*GmRR11d* (Fig. 5b, c). The same result was observed when *proGmNIN1a*:*GUS* was co-expressed with *35* *S*:*GmRR11d* in the transgenic hairy roots of soybean compared with the empty vector (Fig. 5d), suggesting that GmRR11d is indeed a transcriptional repressor of *GmNIN1a*.

Type-B RR transcription factors can directly bind and transactivate target genes^49,50^. Next, we analyzed a 3 kb promoter sequence of *GmNIN1a* and found that there are 7 B-type RR binding sites and 2 putative ARR11 binding sites (Supplementary Fig. 14a), indicating that GmRR11d may directly bind to the *GmNIN1a* promoter and repress its expression. To test this hypothesis, we first conducted chromatin immunoprecipitation (ChIP) assays in soybean hairy roots at 6 DAI. We found that GmRR11d was significantly enriched at the promoter region containing two B-type RR binding sites GGATT of *GmNIN1a* (Fig. 5e, f and Supplemental Fig. 14a). To validate the direct binding of GmRR11d to the specific region of the *GmNIN1a* promoter, we next performed a protein-DNA pull-down assay. For this experiment, we expressed and purified the full-length GmRR11d protein fused to maltose-binding protein (MBP), MBP-GmRR11d, in *Escherichia coli*. Indeed, MBP-GmRR11d was able to interact with DNA fragment containing GGATT (Supplementary Fig. 15). To confirm that GmRR11d represses *GmNIN1a* expression through the specific binding to the B-type RR binding sites in the promoter of *GmNIN1a*, we tested the binding activity of GmRR11d on the fragment containing the mutated GmRR11d binding site (AAAAA, mprobe) and the transcriptional activity of GmRR11d on the *GmNIN1a* promoter containing the mutated GmRR11d binding sites (*proGmNIN1a-DM*). The binding ability of GmRR11d on the mutated fragment was completely inhibited (Supplementary Fig. 15), as a consequence the transcriptional *GmNIN1a* repression by GmRR11d was completely blocked when *proGmNIN1a-DM*:*GUS* was co-expressed with *35* *S:GmRR11d in N. benthamiana* leaves and in hairy roots (Fig. 5b–d). These results suggest that GmRR11d repression of *GmNIN1a* transcription plays a crucial role in the regulation of *GmNIN1a* and symbiotic nodulation in soybean.

In addition to *GmNIN1a*, three additional orthologous *NIN* genes (*GmNIN1b*, *GmNIN2a*, and *GmNIN2b*) can be found in the soybean genome, which are also induced by rhizobia and mediate nodulation^51^. We then analyzed the expression levels of these *GmNINs* in *35* *S*:*GmRR11d* or *Cas9-GmRR11d* hairy roots. All tested genes displayed a similar expression pattern to *GmNIN1a*, with all genes showing increased expression levels in *GmRR11d* knockout roots while reduced expression levels in *GmRR11d* overexpression roots (Supplemental Fig. 13f, g). These results indicate that GmRR11d may repress the expression of all these *GmNIN* genes. To prove this possibility, we analyzed the promoter sequences of the other three *GmNIN* genes and found many B-type RR binding sites in *GmNIN1b, GmNIN2a* and *GmNIN2b* promoter sequences (Supplemental Fig. 14a). Further ChIP-qPCR assays verified that GmRR11d can bind to the B-type RR binding sites on the promoters of these three *GmNINs* (Supplemental Fig. 14b). Taken together, these results demonstrate that GmRR11d can bind to and repress the expression of all the *GmNIN* genes during nodulation.

### GmRR11d inhibits the transcriptional activity of GmNSP1a

**Fig. 6:**
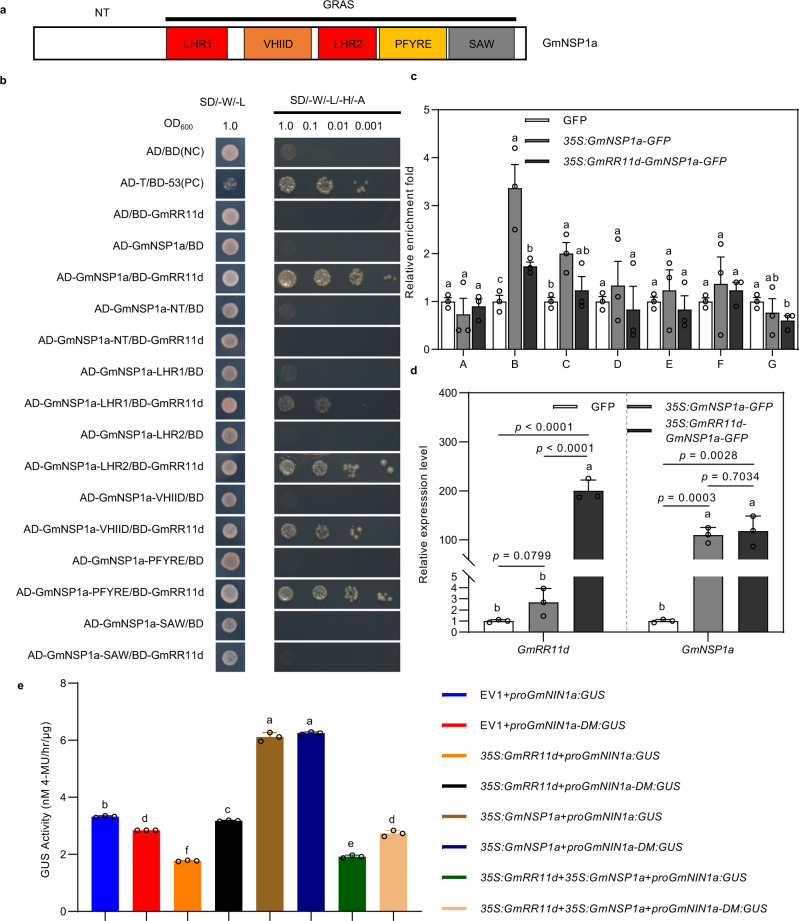
GmRR11d interacts with GmNSP1a and inhibits its transcriptional activity. **a** Domain structure of GmNSP1a. **b** Domain mapping for the interactions between GmNSP1a or its derivatives and GmRR11d by Y2H assay. Three independent experiments were performed with similar results. **c** ChIP assay showing binding of GmNSP1a to *GmNIN1a* promoter and the inhibition effect of GmRR11d to the binding of GmNSP1a to *GmNIN1a* promoter. DNA fragments were co-immunoprecipitated with anti-GFP antibody from chromatin suspensions prepared from *35* *S*:*GmNSP1a-GFP*, *35* *S*:*GmRR11d-GmNSP1a-GFP* or control (empty vector) samples. DNA fragments corresponding to the regions indicated in Fig. 5e. Data are presented as means ± SD of three biological repeats. Different letters indicate significant differences at *P* < 0.05 (One-way ANOVA). **d** The expression level of *GmRR11d* and *GmNSP1a* in hairy root expressing *35* *S*:*GmNSP1a-GFP* or *35* *S*:*GmRR11d-GmNSP1a-GFP*. Data are presented as means ± SD of three biological repeats. More than 30 hairy roots were analyzed in three independent biological repeats. Different letters indicate significant differences at *P* < 0.05 (One-way ANOVA). **e** GUS activity of *proGmNIN1a*:*GUS* and *proGmNIN1a-DM*:*GUS* with different combinations with *35* *S*:*GmRR11d*, *35* *S*:*GmNSP1a* and *35* *S*:*GmRR11d* + *35* *S*:*GmNSP1a* in *N. benthamiana* leaves. Data are presented as means ± SD from three biological repeats. Different letters indicate significant differences at *P* < 0.05 (One-way ANOVA).

The fact that GmRR11d and GmNSP1a antagonistically regulate *GmNIN1a* transcription prompted us to investigate the underlying molecular mechanism. To this end, we first performed protein domain mapping experiments to determine how GmRR11d and GmNSP1a interact using Y2H assays. Interestingly, we found that the C-terminus (CT) of both GmRR11d and GmNSP1a was sufficient for their interaction in *S. cerevisiae* (Supplementary Fig. 16, Fig. 6a, b). Notably, the C-terminus of GmRR11d has no conserved domain, while the C-terminus of NSP1a is a highly conserved GRAS domain consisting of five motifs, including LHRI, VHIID, LHRII, PFYRE, and SAW that are essential for its interaction with NSP2 and among them, the LHR1 and LHR2 motifs are responsible for the DNA binding activity of NSP1^48^. To confirm which domain of the GmNSP1a C-terminus binds to GmRR11d, we further truncated the GRAS domain into LHR1, VHIID, LHR2, PFYRE, and SAW motifs and tested their interactions with GmRR11d. The result showed that all motifs except SAW interacted with GmRR11d (Fig. 6a, b). The interaction between GmRR11d and GmNSP1a likely disrupts the ability of GmNSP1a to form a complex with GmNSP2 and its ability to bind to the DNA, such as binding to the *GmNIN1a* promoter.

To verify the effect of GmRR11d on the DNA binding of GmNSP1a, we conducted ChIP and EMSA assays. The ChIP assay confirmed that GmNSP1a was enriched in regions B and C of the *GmNIN1a* promoter, while the enrichment of GmNSP1a on *GmNIN1a* promoter was significantly reduced in the presence of GmRR11d (Fig. 6c, d). Furthermore, our EMSA results also confirmed that the binding of GmNSP1a to the *GmNIN1a* promoter was negatively correlated with GmRR11d (Supplementary Fig. 17). Moreover, we performed GUS assays to validate the inhibitory role of GmRR11d on GmNSP1a activation of *GmNIN1a* transcription in vivo. GUS activity analysis showed that GmNSP1a can activate both wild type and the mutated (*proGmNIN1a-DM*) *GmNIN1a* promoters, but this activation was effectively suppressed by GmRR11d in *N. benthamiana* leaf cells (Fig. 6e, Supplemental Fig. 18). These results confirm the negative role of GmRR11d on GmNSP1a transcriptional activity and suggest that the binding of GmRR11d on *GmNIN1a* promoter is not essential for the inhibition of GmNSP1a-activated *GmNIN1a* expression. Thus, we conclude that GmRR11d interaction with GmNSP1a is sufficient to interrupt the binding of GmNSP1a to the *GmNIN1a* promoter.

### GmRR11d functions downstream of GmNARK to regulate nodule number

**Fig. 7:**
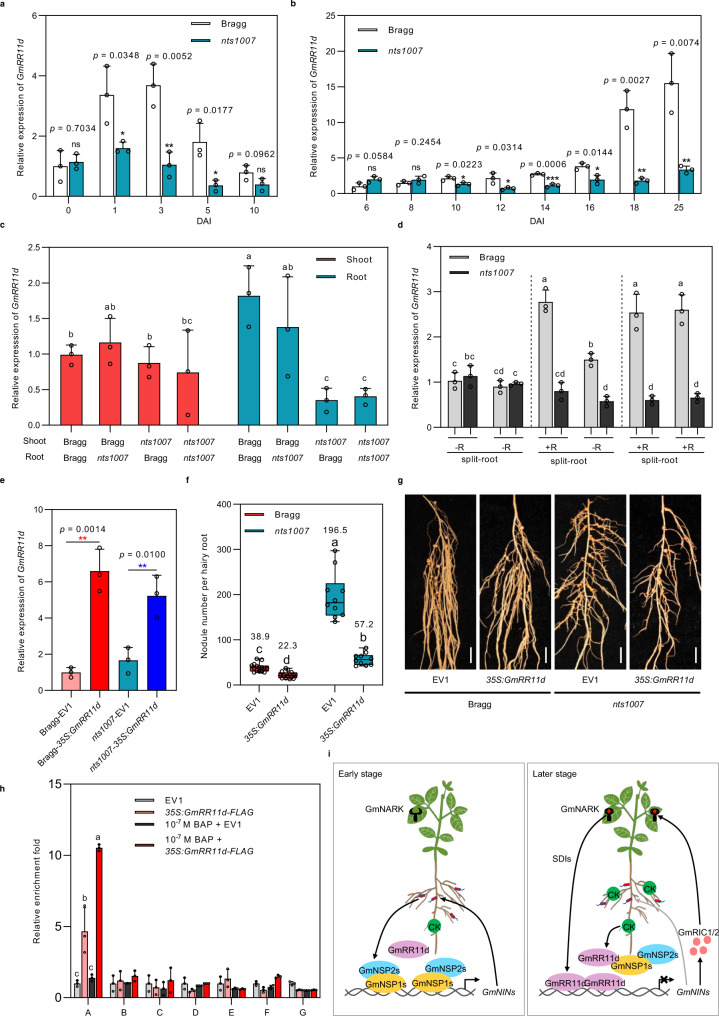
GmRR11d acts as a downstream regulator of GmNARK in soybean. **a**, **b** The expression of *GmRR11d* in roots **a** and nodules **b** of wide-type cv Bragg and *GmNARK* mutant *nts1007*. Seven-day-old-plants were inoculated with USDA110. The roots were harvested at 0, 1, 3, 5, and 10 DAI and the nodules were harvested at 6, 8, 10, 12, 14, 16, 18, and 25 DAI. Data are presented as means ± SD from three biological repeats. More than 9 samples were analyzed in three independent biological repeats. Asterisks indicate significant differences relative to the wild type control. Two-sided Student’s *t*-test, *p < 0.05; p** < 0.01; ***p < 0.001. **c** The expression of *GmRR11d* in shoots and roots of Bragg/Bragg, Bragg/*nts1007*, *nts1007*/*nts1007* and *nts1007*/Bragg grafting plants. Data are presented as means ± SD from three biological repeats and 6 samples were collected for expression analysis. Different letters indicate significant differences at *P* < 0.05 (One-way ANOVA). **d** The expression of *GmRR11d* in split-roots of Bragg and *nts1007*. -R/-R and +R/ + R treatment as negative and positive control. Data are presented as means ± SD from three biological repeats and 12 roots were collected for expression analysis. Different letters indicate significant differences at *p* < 0.05 (Two-way ANOVA) **e** qRT-PCR analysis of *GmRR11d* in empty vector (EV1) and *35* *S*:*GmRR11d* transgenic hairy roots of Bragg and *nts1007* plants. Data are presented as means ± SD from three biological repeats and more than 10 roots were collected for expression analysis Asterisks indicate significant differences relative to the EV1 control. Two-sided Student’s *t*-test, **p < 0.01. **f** Nodule number of Bragg and *nts1007* plants expressing EV1 and *35* *S*:*GmRR11d* at 21 DAI. Data are presented as means ± SD from three biological repeats and more than 10hairy roots were collected for analysis. Different letters indicate significant differences at *p* < 0.05 (Two-way ANOVA). **g** Phenotypes of nodules from individual hairy roots of Bragg and *nts1007* plants expressing EV1 and *35* *S*:*GmRR11d* at 21 DAI. **h** BAP treatment increases the enrichment of GmRR11d in the promoter of *GmNIN1a*. DNA fragments were co-immunoprecipitated with anti-FLAG antibodies from chromatin suspensions prepared from *35* *S:GmRR11d-FLAG* or control (EV1) samples treated with or without 10^−7^ M BAP. The DNA fragments were normalized to the input data. Data are presented as means ± SD from three biological repeats. Different letters indicate significant differences at *p* < 0.05 (one-way ANOVA). **i** A proposed working model for GmRR11d-mediated nodulation inhibition. At low nitrogen conditions, rhizobia infection induces GmNSP1a to activate *GmNIN1a* that promotes nodulation and autoregulation of nodulation (AON). At the early infection stage, the level of GmRR11d is low and CK level is suitable for nodulation, GmNSP1s complexes with GmNSP2 to activate *GmNIN* expression; activation of AON induces expression of B-type regulator GmRR11d which interacts with GmNSP1a and suppresses its transcriptional activation of *GmNIN1a*. Meanwhile, AON-induced CK accumulation enhances binding of GmRR11d to the *GmNIN1a* promoter to repress its expression induced by CK and to activate CK signaling, thereby inhibiting nodulation of soybean.

Our results that *GmRR11d* was induced by rhizobia and reached its highest expression level at the onset of nodule formation but negatively regulated nodule number led us to hypothesize that *GmRR11d* may be upregulated by AON to inhibit further nodule formation. To test this hypothesis, we first assessed the effects of GmNARK on the transcription levels of *GmRR11d*. *GmRR11d* was induced by rhizobia and gradually increased as nodule development in roots of the wild-type (*Glycine max*) cv. Bragg (Fig. 7a, b), which is similar to the pattern in Williams 82. In contrast, transcriptional induction of *GmRR11d* in the infected roots was almost completely blocked in the knockout mutant of *GmNARK* (supernodulating *nitrate-tolerant symbiotic 1007*, *nts1007*) (Fig. 7a, b). The results suggest that rhizobial induction of *GmRR11d* is likely to be dependent on the function of GmNARK.

To confirm that the failure of *GmRR11d* induction in *nts1007* is caused by the function of *GmNARK* in shoots, we analyzed the expression of *GmRR11d* in grafted plants. Indeed, the level of *GmRR11d* expression was reduced when the plants were grafted with *nts1007* mutant scions (Fig. 7c). We further tested the expression of *GmRR11d* in a split-root system of the cultivar Bragg and *nts1007*. qRT-PCR and GUS activity assays showed that *GmRR11d* expression was induced significantly in non-inoculated part compared with control in Bragg, but the induction of *GmRR11d* was blocked completely in *nts1007* (Fig. 7d, Supplemental Fig. 19). These results demonstrate that GmNARK in shoots plays a key role in *GmRR11d* induction during nodulation.

To determine the genetic relationship between *GmNARK* and *GmRR11d*, we overexpressed *GmRR11d* in wild-type Bragg and *nts1007* mutant roots and evaluated the nodule number of the composite transgenic plants. qRT-PCR showed that the expression level of *GmRR11d* was substantially elevated in the transgenic hairy roots of both the wild type and *nts1007* (Fig. 7e). Overexpression of *GmRR11d* significantly reduced the number of root nodules in the wild type (Fig. 7f, g), which is consistent with the results in Williams 82 (Fig. 4). The *nts1007* mutant indeed exhibited a supernodulating phenotype, but interestingly, the number of nodules per transgenic hairy root of *nts1007* overexpressing *GmRR11d* was dramatically decreased, and the supernodulating phenotype of the *nts1007* mutant was almost completely rescued (Fig. 7f, g). These results indicate that GmNARK may inhibit nodulation in shoots by inducing *GmRR11d* expression in roots. To further strengthen the relationship between *GmRR11d* and AON, we analyzed *GmRR11d* expression in the hairy roots overexpressing *GmRIC1* and *GmRIC2*. As expected, *GmRR11d* was upregulated in the hairy roots overexpressing *GmRIC1 and GmRIC2* (Supplementary Fig. 20), supporting the notion that *GmRR11d* acts downstream of AON. Unexpectedly, the expression levels of *GmRIC1* and *GmRIC2* was upregulated in hairy roots overexpressing *GmRR11d* and downregulated in hairy root knockout *GmRR11d* (Supplementary Fig. 21), suggesting that GmRR11d also regulates AON through positive feedback.

*TOO MUCH LOVE* (*TML*), which encodes a F-box protein and acts downstream of HAR1 is required for nodulation inhibition in *L. japonicus*^52^. To explore the possible relationship between GmRR11d and GmTML1b, a downstream negative regulator of AON, we measured the transcription level of *GmRR11d* in the hairy roots overexpressing *GmTML1b* and found that the level of *GmRR11d* expression remained unchanged (Supplementary Fig. 20). Furthermore, we found that *GmTML1b* expression was not altered in *GmRR11d* knockout roots compared with the vector control roots, although it was slightly upregulated in the hairy roots overexpressing *GmRR11d* (Supplementary Fig. 21). These results suggest that GmRR11d is a key component in the AON signalling but inhibits nodulation in a mainly GmTML1b-independent manner.

### Cytokinin promotes the binding of GmRR11d to the *GmNIN1a* promoter

It has been shown that the transcription levels of B-type *RR* genes are not responsive to CKs, but the binding of type-B RR proteins to its target genes can be enhanced by CKs in *Arabidopsis*^30^. Unlike the *Arabidopsis* B-type *RR* genes, *GmRR11d* was induced by various concentrations of CKs (Fig. 2a), we next wondered whether CKs can also enhance GmRR11d binding to the *GmNIN1a* promoter. To answer the question, we expressed *35* *S:GmRR11d-FLAG* in hairy roots of soybean in the absence or presence of CKs and performed a ChIP assay. The analysis result revealed that CK treatment promoted GmRR11d binding to the promoter region of *GmNIN1a*. In the absence of exogenous CKs, there was a low level of enrichment of GmRR11d at the binding site on the *GmNIN1a* promoter, but in the presence of CK treatment, there was a substantial increase in enrichment of GmRR11d on the *GmNIN1a* promoter region (Fig. 7h). This result indicates that cytokinin can enhance GmRR11d binding to the *GmNIN1a* promoter.

## Discussion

Maintaining the optimal nodule number in legumes requires coordination of nodulation and autoregulation of nodulation. When legume plants detect low nitrogen levels, they attempt to form root nodules by activating *NIN* through CKs^34,37,53^. Subsequently, activation of the AON pathway can alleviate CK-activated nodulation by suppressing *NIN* expression in roots^26,46^. Thus, the regulation of the CK-NIN axis is essential for the best outcomes in the trade-off between plant growth and symbiotic nodulation. Here, we found that the CK signaling type-B transcription factor GmRR11d is induced by rhizobia through AON signaling. *GmRR11d* is induced by CKs and upregulation of *GmRR11d* causes CK hypersensitivity of plants. We also found that GmRR11d negatively regulates nodulation by repressing transcription of *GmNIN1a* which is enhanced by CK, and by suppressing transcriptional activation activity of GmNSP1a on *GmNIN1a*. Our work reveals a novel mechanism that AON signaling maintains nodulation homeostasis through simultaneous induction of CK hypersensitivity and attenuation of GmNIN1a-mediated NF signaling during nodulation.

Induction of transient CK biosynthesis and accumulation in the root susceptible zone is essential for nodule initiation^23,33^. However, excessive CK accumulation or exogenous CK treatments inhibits root nodulation^24,54^. In *Lotus japonicus*, AON signaling can induce overproduction of CKs in the shoot that transports to the infected root to suppress nodulation^26,33^. In addition, it has been shown that shoot control of root nodulation is at downstream of CK signaling-mediated activation of nodule initiation^33,47^. It is apparent that the regulation of the levels of CKs and/or CK response is central for nodulation homeostasis. It is likely that the NF and AON signaling pathways converge on, and dynamically regulate the levels and sensitivity of cellular CKs to control nodulation homeostasis in legumes. In this work, we provide evidences that AON can induce a type-B RR transcription factor in infected root that connects the AON, NF and CK signaling pathways to inhibit nodulation. Our results showed that a type-B RR transcription factor encoding gene *GmRR11d* is induced by rhizobia in soybean roots at the time when the AON signaling is switched on and its expression induction is largely dependent on the shoot GmNARK. In addition, *GmRR11d* expression is regulated by GmRIC1 and GmRIC2. Importantly, GmRR11d negatively regulates soybean nodulation in a GmNARK-dependent manner. Thus, GmRR11d is a key downstream component of the long-distance negative feedback loop during nodulation. It has been shown that TML targeted by miR2111 is a central factor at the downstream of AON in *L. japonicus*^52,55^. However, our data showed that GmRR11d and GmTML1b may suppress nodulation independently of each other in soybean. It is likely that GmRR11d and GmTMLs achieve the same out-put through different mechanisms in soybean.

In *L. japonicus* and soybean, NIN activates the expression of *CLE-RS1/RS2* and *GmRIC1/GmRIC2* to turn on AON, which in turn leads to reduced expression of *NIN* and nodulation inhibition^41,46^. Decreased expression of *NIN* should lead to downregulation of *CLE-RS1/RS2* and *GmRIC1/GmRIC2*, thus attenuating AON as well. However, we found that although GmRR11d represses *GmNIN1a* expression, the expression of *GmRIC1* and *GmRIC2* in *35* *S:GmRR11d* roots increased rather than decreased. This is conceivable because AON is a continuous process that must occur during nodule formation and development. Given that *GmRR11d* and *GmRIC1/2* genes have overlapping expression pattern in infection and nodule development^56^ and there are GmRR11d binding sites in the promoters of *GmRIC1*/*2* (Supplementary Fig. 22), it is likely that GmRR11d can directly activate the expression of *GmRIC1*/*2* in a spatio-temporal manner. The fact that B-type RR transcription factors can regulate expression of a wide range of genes^30,57^ supports our hypothesis. Further analyses of direct binding and activation of GmRR11d on *GmRIC1* and *GmRIC2* promoter would prove our hypothesis. Nodulation and nodulation inhibition are complex processes involving various hormones and multiple signaling pathways. Recently, GA was shown to activate AON through activation of *NIN*, suggesting more regulatory pathways that induce *CLE-RS2* and *GmRIC1/2* expression and activate AON^58^. B-type RRs can target various genes including the genes in hormone synthesis, metabolism and signal transduction^57^. Interestingly, all three important genes *GmRR11d*, *GmRIC1,* and *GmRIC2* in AON are induced by CKs, suggesting a crucial role of CKs in autoregulation of nodulation, but we don’t exclude the possibility that GmRR11d activates *GmRIC1* and *GmRIC2* through other mechanisms. Based on these findings, we proposed that nodulation autoregulation is continuously activated and dynamically modulated during nodule development to maintain nodulation homeostasis although AON is activated early with nodule formation.

Type-B RRs are indispensable for CK signal transduction and increasing the expression level of the genes results in hypersensitivity to CKs and altered growth and development in *Arabidopsis*^29^. Our results demonstrate that GmRR11d is a functional type-B RR transcription factor. It contains both receiver and Myb-like domains that are also found in other type-B RR proteins, and has DNA binding activity, and is localized in the nucleus. Our results also proved that GmRR11d mediates the activation of CK signal transduction as the increasing level of *GmRR11d* increases the sensitivity of soybean roots to CKs through induction of type-A *RR* genes, such as *GmRR15a* and *GmRR15b*. It is worthy to note that unlike other type-B ARRs in *Arabidopsis* (e.g., ARR10)^30^, the *GmRR11d* promoter contains RR DNA-binding sites and the expression of *GmRR11d* is induced by CKs; but like the type-B ARRs in *Arabidopsis*^30^, GmRR11d binding to its target DNA is also enhanced by low-level CKs. The results suggest that GmRR11d uses a conserved mechanism for mediating CK response of soybean. The induction of *GmRR11d* expression and increased DNA binding activity by CKs may result in greater transcriptional activity of GmRR11d in the regulation of CK responsive genes, such as *GmRR15s* and CK hyposensitivity that affects cell division and expansion^30^. Together with observations that AON induces *GmRR11d* expression in rhizobial infection and in nodule formation to inhibit nodulation, it is conceivable that AON signaling may also activate GmRR11d to induce CK hypersensitivity of infected root and developing nodule, thereby inhibiting nodulation. It remains unknown whether AON also induces biosynthesis of active CKs when AON is turned on, leading to induction and activation of GmRR11d to cause CK hypersensitivity and nodulation inhibition. It would be interesting to know whether and how AON activates *GmRR11d*, as well as *GmRIC1* and *GmRIC2*, to exert their inhibitory role during nodule organogenesis and development.

Upon NFs perception, the *NIN* genes are essential for NF signal transduction. The spatial and temporal expression of *NIN*s and their levels are central for nodulation^10,37,41,46,59,60^. *NIN* genes are activated by the NSP1–NSP2 complex in legumes, and NSP1 can directly bind to nodulation genes in *M. trunctula*^48^. Here, we show that GmNSP1s and GmNSP2s also positively regulate soybean nodulation, and GmNSP1a can activate *GmNIN1a* in soybean nodulation. Thus, NSP1/NSP2-mediated activation of *NIN* genes is a conserved mechanism that triggers the nodulation of legumes. Previously, we have shown that AON inhibits nodulation by suppressing the expression of *GmNIN1a* and the downstream genes^41^, but the underlying mechanism is still unknown. In this study, we used a protein–protein interaction approach to identify the GmRR11d as an interacting protein of GmNSP1a that negatively regulates nodulation. We proved that GmRR11d represses *GmNIN1a* expression through direct repression of *GmNIN1a* transcription and suppression of transcriptional activation activity of GmNSP1a on *GmNIN1a* expression. Our results showed that GmRR11d directly binds to the promoter of *GmNIN1a* to repress its transcription. In addition, GmRR11d through its C terminal can interact with the GRAS domain of GmNSP1a that is involved in its interaction with GmNSP2 and its DNA binding to suppress its transcriptional activity on *GmNIN1a*. Importantly, our data that increasing the protein quantity of GmRR11d decreases the ability of GmNSP1a to bind to the *GmNIN1a* promoter supports the hypothesis. Furthermore, we showed that repression of gene transcription by GmRR11d applies to all the *GmNIN* genes. Thus, GmRR11d can repress *GmNINs* expression by antagonizing the master upstream activator GmNSP1a to effectively inhibit nodulation. In addition to GmNSP1 and GmNSP2, CYCLOPS can also activate *NIN* expression during nodule formation^61,62^. It will be interesting to investigate whether GmRR11d interacts with and affects the transcriptional activity of CYCLOPS on the expression of *GmNIN* or other genes.

Taken into account that GmRR11d is induced by CKs and its binding affinity to the promoter of *GmNIN1a* is enhanced by CKs, we proposed that AON may induce CK biosynthesis and *GmRR11d* to suppress NF signaling in inoculated roots. Since other *GmRR11d* paralog genes (*GmRR11a-c*) were differentially expressed during nodulation and these GmRR11s are highly conserved, we do not exclude the possibility that these genes also mediate nodulation inhibition by precise spatial-temporal modulation of CK response of infected root and nodulation activity. Type-B RR transcription factors, such as ARR10 in *Arabidopsis*, can regulate plant growth and development by targeting various genes in CK primary-response pathway and other regulatory signaling pathways^30^. Genome-wide identification of the target genes of GmRR11s will provide the molecular basis of transcriptional inhibition of nodulation and nodule development by AON and CKs.

Legumes have evolved the AON systemic pathway to control the trade-off between plant growth and symbiotic nodulation^39,63^. It is conceivable that multiple mechanisms act to fine tune NF signaling and nodulation, but regardless of CK origin, the regulation of the CK level and CK sensitivity is central for optimal nodulation^26,30,33^. Based on our findings, we propose a model where AON induces GmRR11d through GmNARK during early root nodulation, which then inhibits further nodulation by simultaneously inducing CK hypersensitivity and repressing *GmNIN* expression and NF signaling (Fig. 7i). At the early infection stage, GmNSP1s complexes with GmNSP2 to activate *GmNIN* expression when the level of GmRR11d is low and CK level is suitable for nodulation; while at the later stage of rhizobial infection, GmNSP1s binding to *GmNIN* promoters are effectively suppressed by increasing levels of GmRR11d and CKs at the onset of AON. In the meantime, high amount of GmRR11d can also directly bind to and repress the expression of *GmNINs*. The repression of *GmNINs* attenuates NF signaling activity after AON activation and blocks further rhizobial infection and nodule formation. Because NF, AON, CK biosynthesis and signaling are highly conserved in legumes^39,43–45,48^, our findings thus reveal the transcriptional regulation of *NINs* as a crucial interaction between CKs and NF/AON signaling pathways in the control of nodule number in legumes.

## Methods

### Plant growth, hairy root transformation and rhizobium inoculation

Soybean [*G. max* (L.) Merrill cv. Williams 82, Bragg and the *GmNARK* mutant *nts1007* in Bragg background] plants were used for gene cloning, gene expression and functional analysis of *GmRR11d*, *GmNSP1/2* and *GmNIN1a*. Growth of soybean seedlings, hairy root transformation with *Agrobacterium rhizogenesis* strain K599 and inoculation with *Bradyrhizobium* (*B*.) *diaefficiens* strain USDA110 were conducted as previously described^64,65^. Briefly, soybean seedlings were germinated and grown under 16 h/8 h light/dark conditions in a growth room at 25–26 °C.Young seedlings at 3–4 days after germination were used for hairy root transformation. For nodulation assays, transgenic composite plants were transplanted to pots containing vermiculite. The plants were grown for 1 week to allow rooting and then were inoculated with 30 mL *B. diaefficiens* strain USDA110 suspended in distilled water (OD_600_ = 0.08). Nodule phenotypes were evaluated at 21 DAI or 28 DAI.

### DNA extraction and validation of transgenes

Plant samples harvested from the composite plants were used for DNA extraction using the CTAB method as described previously^66^. Briefly, the hairy roots were ground in the isolation buffer (2% hexadecyltrimethylammonium bromide [CTAB], 1.4 M NaCl, 0.2% 2-mercaptoethanol, 20 mM EDTA, 100 mM Tris-HCl, pH8.0) and incubated at 60 °C for 30 min, DNA was then extracted with chloroform-isoamyl alcohol (24:1), after centrifugation (12000 × *g* for 10 min), the aqueous phase was transferred to clean tubes and the DNA was precipitated by ethanol. Presence of the *Bar* or Green fluorescent protein (*GFP*) gene was validated using PCR, and the hairy roots transformed with the corresponding empty vectors were used as negative controls. For the hairy roots harboring two genes, the presence of both *Bar* and *GFP* genes was detected by PCR. For confirming the edited hairy root in *GmRR11d*, *GmNSP1a*, *GmNSP1b* or *GmNSP2a/b*, the DNA fragments containing the sgRNAs were amplified and sequenced. The primers for the *Bar* and *GFP* genes are listed in Supplementary Table 1.

### RNA extraction and quantitative PCR analysis

Total plant RNAs were extracted using TRIpure Reagent (Aidlab Biotechnologies Ltd., Beijing, China). Genomic DNAs were removed by gDNA Wiper Mix (Vazyme Biotech Co., Ltd., Nanjing, China). cDNAs were produced using a FastQuant RT Kit (Vazyme Biotech Co., Ltd.). qRT-PCR analyses were performed using SuperReal PreMix Plus (Vazyme Biotech Co., Ltd.). The primers used in qRT-PCR are listed in Supplementary Table 1.

### Vectors construction

For the *proGmRR11d:GUS*, *35* *S*:*GmRR11d* and *RNAi-GmRR11d* constructs, the promoter (2000 bp  upstream of ATG) or coding sequence of *GmRR11d* were amplified and cloned into pDONR207 by BP reactions. The resulting entry construct was inserted into pMDC162 (*proGmRR11d*:*GUS*), pEarlygate100-Flag (*35* *S*:*GmRR11d-Flag*) and pK7GWIWG2D(II) (*GmRR11d-RNAi*) through LR reactions. For the *Cas9-GmRR11d*, *Cas9-GmNSP1a*, *Cas9-GmNSP1b* and *Cas9-GmNSP2a/b* constructs, the sgRNAs were designed using CRISPR-P 2.0 software (http://crispr.hzau.edu.cn/CRISPR2/)^67^. The pCBC-DT1T2 vector was used as the template to amplify the fragments with two sgRNAs, and then the fragments were connected to the skeleton vector pKSE401-GFP by Golden Gate reaction by the enzyme *Bsa1*^68,69^. For the *35* *S*:*GmNSP1a-GFP* constructs, the coding sequence of *GmNSP1a* was amplified from cv. Williams 82 and inserted into pTF101-GFP vector using *EcoRI* and *SalI*. For subcellular localization, the coding sequences of *GmRR11d*, *GmNSP1a*, *GmNSP2a* were amplified and inserted into pTF101-GFP vector (*35* *S*:*GmRR11d-GFP*) or pEarlygate101-YFP vector (*35* *S*:*GmNSP1a-YFP* and *35* *S*:*GmNSP2a-YFP)*. For the *35* *S*:*GmRIC1*, *35* *S*:*GmRIC2* and *35* *S*:*GmTML1b* constructs, the coding sequence of *GmRIC1*, *GmRIC2* and *GmTML1b* were amplified and cloned into pDONR207 by BP reactions. The resulting entry construct was inserted into pEarlygate100-Flag through LR reactions.

### Yeast two-hybrid assay

To identify GmNSP1a-interacting proteins, we performed a Y2H screen using the Matchmaker GAL4 Yeast Two-Hybrid System (Clontech, USA), according to the manufacturer’s manual. RNA was extracted from mixture of roots and nodules using the RNeasy Plant Mini Kit (Qiagen) following the manufacturer’s instructions. Total RNA samples with an RNA integrity number >6.7 were used for the Y2H library construction. The cDNA library was constructed by OE Biotech (Shanghai, China).

The full-length coding sequences of *GmRR11d*, *GmNSP1a* and *GmNSP2a* were amplified and the PCR products were cloned into the entry vector pDONR207 by BP reactions. pDONR207 harboring these genes were then ligated into pGBKT7 (BD) or pGADT7 (AD) by LR reactions. Yeast-two hybrid (Y2H) assays were done according to the Matchmaker GAL4 Two-Hybrid System (Clontech, Mountain View, CA). The *β*-Galactosidase activity of yeast cell was following the method of Möckli and Auerbach^70^.

To further verify the interactions between GmRR11d/its domains and GmNSP1a/its domains, GmRR11d/GmNSP1a and their related domains were fused with the BD/AD domain in the pGBKT7/pGADT7 vector individually. For testing interactions, the above constructs were co-transformed into the yeast strain *Saccharomyces cerevisiae* AH109. Transformation was confirmed by growth on SD/-Leu/-Trp (SD/−2) medium. Interactions were assayed by spreading 2.5 μL of suspended transformed yeast on plates containing SD/-Ade/-His/-Leu/-Trp (SD/−4) medium. The interactions were observed after 3-4 days of incubation at 30 °C. The primers used in Y2H assays are listed in Supplementary Table 1.

### BiFC assay

The coding sequences of *GmRR11d*, *GmNIN1a*, *STF1*, *GmNSP1a* and *GmNSP2a* were cloned into the pEARLYGAYE201-YN and pEARLYGAYE202-YC through the Gateway reaction system (Invitrogen, Carlsbad, CA), respectively. The constructs were transformed into *Agrobacterium tumefaciens* strain GV3101 for infiltration transformation of *Nicotiana* (*N*.) *benthamiana* leaves. YFP fluorescence was detected using a Leica confocal laser scanning microscope (Leica Microsystems). For visualizing nuclei, leaves were stained with 2 mg/mL 4′, 6-diamidino-2-phenylindole (DAPI) for 10 min in dark conditions before observation.

### Localized surface plasmon resonance

Protein-protein interactions were measured using an OpenSPR LSPR biosensor (Nicoya Life Science, Kitchener, Canada), as described previously^71^. In brief, 200 mL (50 μg/mL) of GST-GmRR11d was immobilized on a COOH sensor chip (Nicoya #SEN-AU-10012-COOH) at a flow rate of 20 mL/min in 1× PBS buffer (pH 7.4, RNase-free) and 0.1% (v/v) Tween 20. Free activated carboxyl groups were deactivated with the addition of 200 mL of blocking buffer (Nicoya). The immobilized protein was washed with running buffer (1× HEPES pH 7.4, 0.005% Tween 20) to reach a stable baseline. Buffer matched recombinant MBP-GmNSP1a (25 nM, 50 nM, 100 nM, 200 nM, 400 nM, 800 nM) or MBP-GmNSP2a (0.2 μM, 0.4 μM, 0.8 μM, 1.6 μM, 3.2 μM) was injected into the flow cell at a rate of 100 μL/min. Following a 5 min interaction time, the dissociation was recorded for an additional 7 min. Kinetic binding analysis was performed with the TraceDrawer software package (Ridgeview Instruments, Uppsala, Sweden). Sensorgram traces were fit to a 1:1 Langmuir model to derive affinity (*Kd*) constants.

### Pull-down assay

The full-length coding sequence of GmRR11d was amplified and inserted into the pMAL-c2X vector (MBP-GmRR11d) (New England Biolabs). The full-length coding sequences of GmNSP1a and GmNSP2a were cloned into the pGEX-4T-1 vector (GST-GmNSP1a and GST-GmNSP2a) (GE Healthcare). The GmRR11d fused with MBP and GmNSP1a/GmNSP2a fused with glutathione S-transferase (GST) were expressed in *Escherichia coli* strain BL21 cells at 37 °C for 3 h and induced by 1 mM Isopropyl β-D-1-thiogalactopyranoside (IPTG). MBP-GmRR11d and GST-GmNSP1a/GmNSP2a were purified using amylose resin (New England Biolabs) or Pierce^TM^ Glutathione Agarose (Thermo Fisher Scientific), respectively, according to the manufacturer’s instructions. Pull-down assays were performed as described previously^72^.

The DNA-protein pull-down assay was performed as described by Vert et al.^73^, the biotinylated *GmRR11d* promoter was incubated with streptavidin MagBeads (GenScript, L00424) in TES buffer (10 mM Tris-HCl pH 7.5, 1 mM EDTA pH 8.0, 2 M NaCl) for 1 h at room temperature and then was washed three times in IP100 buffer (100 mM potassium glutamate, 50 mM Tris-HCl pH 7.6, 2 mM MgCl_2_, 0.05% v/v Nonidet P40). The MBP or MBP-GmRR11d was added to DNA-bound beads, and the mixture was rotated for 2 h at 4 °C. The beads were washed three times with IP100 buffer. The proteins were removed from the beads by boiling in 2× SDS loading buffer and subjected to PAGE.

### Co-IP assay

The GmRR11d-MYC construct was co-expressed with GmNSP1a-HA construct in *N. benthamiana* leaves. Tobacco total proteins were extracted in extraction buffer (50 mM Tris-HCl, pH 7.5, 150 mM NaCl, 1 mM EDTA, 10% glycerol, 1% Triton X-100, 1 mM PMSF and 1× protease inhibitor cocktail and 10 μM ΜG132) and then were incubated with 8 µL anti-MYC agarose beads (SIGMA, A7470) for 2 h at 4 °C. The beads were washed five times with extraction buffer, and the immunoprecipitated proteins were eluted with SDS loading buffer with boiling for 5 min for western blotting. Samples were analyzed by western blot with anti-HA (SIGMA, SAB4300603, 1:2500) and anti-MYC (SIGMA, C3956, 1:2000).

### ChIP-qPCR assay

Two grams of transgenic roots expressing *35* *S*:*GmRR11d-FLAG*, *pEarlygate100-FLAG*, *35* *S*:*GmNSP1a-GFP*, *35* *S*:*GmRR11d*-*GmNSP1a-GFP* and *35* *S:GFP* were used for ChIP assays. Roots were cross-linked with 1% formaldehyde at 4 °C for 10 min and neutralized with 0.125 M glycine. Roots were ground to fine power in liquid nitrogen and the nuclei isolated. Immunoprecipitation was done using anti-GFP (ab290, Abcam) or anti-FLAG (A2220, ANTI-FLAG M2 Affinity Gel, Sigma) antibodies. Chromatin precipitated without the use of antibodies was used as a negative control, while chromatin isolated before precipitation was used as an input control. qPCR analysis was performed, and soybean *ELF1b* (*GmELF1b*) was used as an internal control. The specific primers used in this experiment are listed in Supplementary Table 1. Three independent biological repeats were performed for each analysis^41^.

For the effect of exogenous cytokinin treatment on the binding activity of GmRR11d on the *GmNIN1a* promoter, two grams of transgenic roots (10 DAI) expressing EV1 and *35* *S*:*GmRR11d-FLAG* were treated with 0.1 μM 6-benzylaminopurine (BAP) for 4 h.

### The electrophoretic mobility shift assay

The Electrophoretic Mobility Shift Assay (EMSA) was performed as described previously^41^. MBP, MBP-GmRR11d, GST, GST-GmRR11d, and GST-GmNSP1a proteins were expressed in *E. coli* cells and purified. EMSA assay was performed using Light Shift Chemiluminescent EMSA Kit (Pierce, Rockford, IL) according to the manufacturer’s protocol. The binding activity of the protein was analyzed using an oligonucleotide labeled with biotin at the 5′ end (Sangon Biotech). 200-fold unlabeled probe as a competitor were added to the reactions. For GmRR11d, the mutated probe sequence was changed from GGATT to AAAAA. The primers used in EMSA are listed in Supplementary Table 1.

### Transcriptional repression of *GmNIN1a* by GmRR11d in plant cells

*A. tumefaciens* strain GV3101 and *A. rhizogenesis* strain K599 were used for infiltration transformation of *N. benthamiana* leaves and hairy root transformation, respectively. The promoter of *GmNIN1a* was cloned into pCambia1391 vector to generate the reporter constructs *proGmNIN1a*:*GUS* (−2500 bp). The hairy roots expressing *proGmNIN1a*:*GUS* or *proGmNIN1a-DM:GUS* transformed with EV1 or *35* *S*:*GmRR11d* were inoculated with rhizobia for 3 days. For transiently co-expressing *GmRR11d* and *GmNIN1a* genes in *N. benthamiana* leaves, mixed culture with an equal volume (1:1) of each culture were used to inject the back of the leaves (the final OD_600_ was 0.4 for each). At 48 h after injection, GUS expression was analyzed by GUS staining^61^.

### DNA binding activity analysis of GmNSP1a to the promoter of *GmNIN1a*

The constructs *proGmNIN1a*:*GUS* or *proGmNIN1a-DM:GUS* were transformed with EV1, *35* *S*:*GmRR11d*, *35* *S*:*GmNSP1a* and *35* *S*:*GmRR11d* + *35* *S*:*GmNSP1a* into *N. benthamiana* leaves. At 36 h after injection, GUS expression was analyzed by GUS staining.

For *β*-glucuronidase (GUS) enzyme activity, *N. benthamiana* leaves were placed in a mortar containing liquid nitrogen, crushed, and then added with GUS enzyme extraction buffer (10 μL/mg tissue). The supernatant after centrifugation (12000 × g, 4 °C, 10 min) is the extract of GUS enzyme. The flourimetric assay of GUS activity were carried out by the methods, as described previously^74^. In this experiment, protein concentrations were determined by using the Bradford’s dye binding assay^75^.

### Exogenous application of 6-benzylaminopurine

For *GmRR11d* and *GmRR15a/b* expression in response to exogenous CK application, soybean seeds (W82) were germinated in vermiculite irrigated once with a low-nitrate solution. Seven-day-old seedlings were treated with 6-benzylaminopurine (BAP) using different concentrations for 3 h. PBS buffer was used as a control for analyzing the expression of *GmRR11d* and *GmRR15a/b*. To detect whether the expression of *GmRR11d* affects the sensitivity of soybean to cytokinin, empty vector (EV1) and *35* *S:GmRR11d-FLAG* vectors were used to hairy root transformation with *A. rhizogenesis* strain K599 using different concentrations (0, 0.001, 0.1, and 1 μM) of BAP.

### Grafting experiments

The Bragg and *nts1007* seeds used as scion and rootstock were seeded in vermiculite for germination (16 h light/8 h dark, 26 °C, 60% relative humidity). Ten-day-old seedlings were cut at the hypocotyl and grafted to reciprocal rootstock using 4 cm parafilm, then sealed with plastic film and cultured in dark condition for 3-4 days. It can be confirmed that the grafted seedlings survived when the first young trifoliate leaves show obvious signs of growth, then inoculated with *B. diazoefficiens* USDA110 and the shoots and roots were harvested at 4 days after inoculation.

### Histochemical analysis

The infected hairy roots and nodules of soybean composite plants expressing *proGmRR11d*:*GUS* were stained with X-Gluc to test *β*-glucuronidase activity at the specified time points.

### Split-root assay

The split-root experiments were carried out by the methods as described previously^76^. The seven-day-old Bragg and *nts1007* roots were divided into two parts and treated with (+R) or without (-R) *B. diazoefficiens* strain USDA110. The roots (-R/-R, + R/-R, + R/ + R) were collected at 3 DAI and test the expression of *GmRR11d*. For the GUS staining of *proGmRR11d:GUS* in hairy root transformation system, the hairy roots were divided into two parts and treated with or without *B. diazoefficiens* strain USDA110. The hairy roots (-R/-R, + R/-R) were collected at 3 DAI.

### Phylogenetic analysis

The protein sequences of *G. max*, *M. truncatula* and *A. thaliana* were obtained from Phytozome v12.1 database (https://phytozome.jgi.doe.gov/)^77^ and *L. japonicus* protein sequences were obtained from Miyakogusa.jp 3.0 database (http://www.kazusa.or.jp/lotus/) and then imported into MEGA7.0^78^ for complete alignment using Explorer/Clustal^79^. The phylogenetic tree was built using MEGA7.0. Phylogenies were built using the neighbor-joining method with the bootstrapping value set at 1000 replications.

### Bioinformatics analysis

The protein sequences were further characterized through a multiple sequence alignment via Clustal Omega hosted by EMBL-EBI (https://www.ebi.ac.uk/Tools/msa/clustalo/). Synteny analysis was analyzed using TBtools software^80^. The gene structure analysis of *GmNSP2a/b* and the homologous genes were performed using GSDS 2.0 software^81^. For protein alignment of ARR7 and ARR15 with their orthologs in soybean, protein sequences of GmRR15a/b/c/d, ARR7 and ARR15 were aligned using CLUSTALW website (https://www.genome.jp/tools-bin/clustalw) and visualized by ESPript 3.0 website (http://espript.ibcp.fr/ESPript/cgi-bin/ESPript.cgi)^82^.

### Statistical analysis

Data was analyzed using GraphPad Prism 8 (GraphPad Software, Inc., La Jolla, CA). Statistical significance was determined by ANOVA with Tukey’s post hoc test or the two-tailed Student’s *t*-test. The type of statistical significance test was described in the figure legends.

### Reporting summary

Further information on research design is available in the Nature Portfolio Reporting Summary linked to this article.

## Supplementary information

Supplementary Information

Reporting Summary

## Data Availability

All data are available in the main text and/or supplementary materials. The source data for Figs. 17, Supplementary Figs. 1, 5, 7, 9, 11–15, 17, 20–21 are provided as a Source Data file. Source data are provided with this paper.

## Source data

Source Data
