# Supplementary material for: The B-type response regulator GmRR11d mediates systemic inhibition of symbiotic nodulation
Source: Nat Commun. 2022 Dec 10;13:7661. doi: 10.1038/s41467-022-35360-9 (PMC9741591; doi:10.1038/s41467-022-35360-9)
Supplement: Supplementary file 1 — Supplementary Information [file 41467_2022_35360_MOESM1_ESM.pdf]

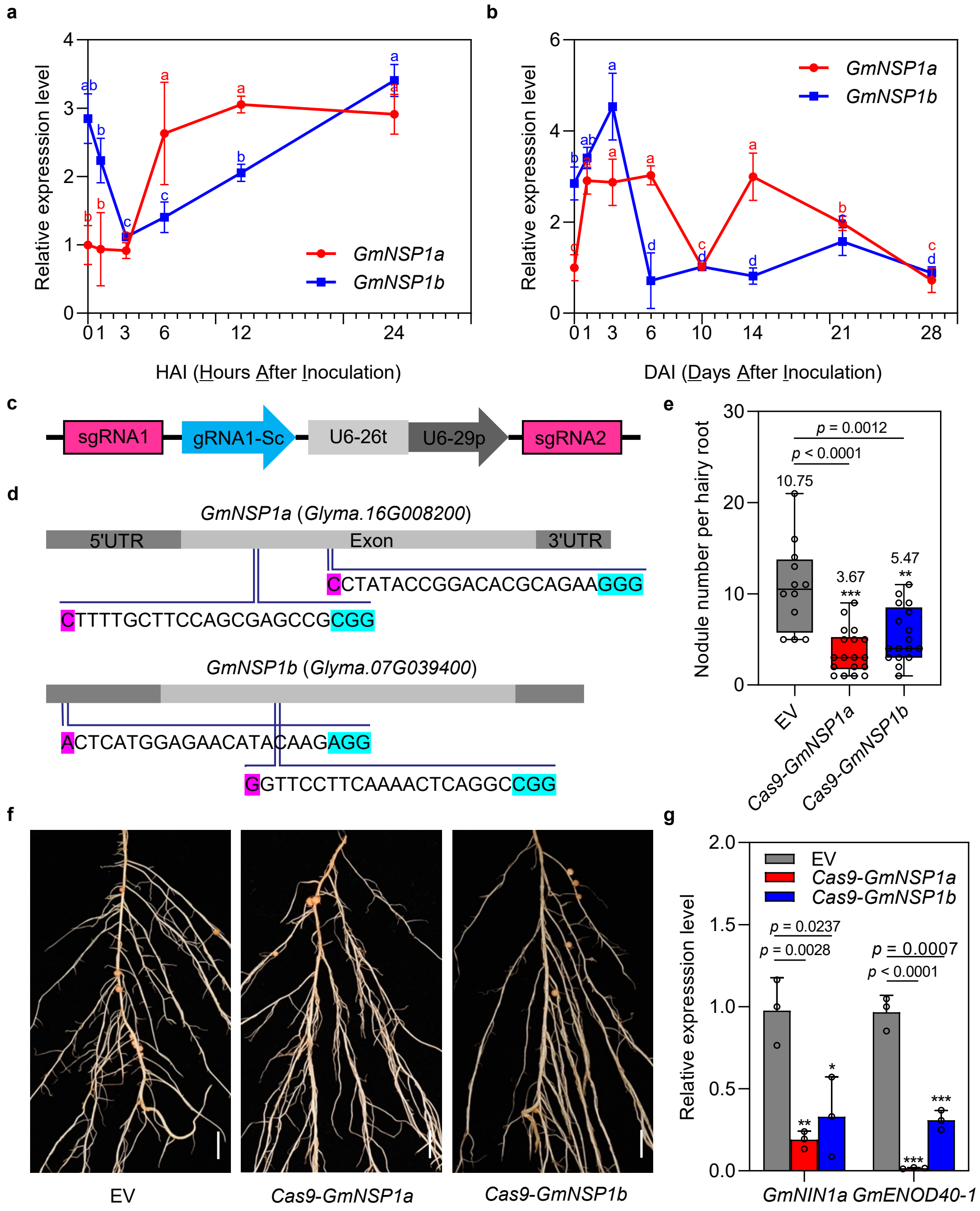

Supplementary Figure 1. *GmNSP1a* and *GmNSP1b* are *NSP1* homologs.

(a-b) The expression pattern of *GmNSP1a* and *GmNSP1b* by qRT-PCR. Seven-day-old seedlings were inoculated with *B. diazoefficiens* strain USDA110 and the infected roots were collected at 0, 1, 3, 6, 12, 24 hours after inoculation (HAI) (a) or 0, 1, 3, 6, 10, 14, 21, 28 days after inoculation (DAI) (b) for the expression of *GmNSP1a/b*. *GmELF1b* was used as endogenous control gene. Data are shown as the means ± SD from three biological replicates. Different letters indicate significant differences at $p < 0.05$ (One-way ANOVA). (c) Schematic diagram of pKSE401-GFP vector construction. (d) The diagram of two sgRNAs (small guide RNAs) sequences of *GmNSP1a* and *GmNSP1b*. (e) Quantitative analysis of the nodule number per hairy root expressing empty vector (EV) (n = 12), *Cas9-GmNSP1a* (n = 18) and *Cas9-GmNSP1b* (n = 17). More than 32

hairy roots for each construct were tested by sequencing and only the homozygous edited hairy roots were calculated. Nodule numbers were determined at 21 DAI. Data are shown as means ± SD, and asterisks indicate significant differences relative to EV control. Two-sided Student's *t*-test, $**p < 0.01$; $***p < 0.001$. Boxes indicate the first and third quartiles and the whiskers indicate the minimum and maximum values, the black lines within the boxes indicate the median values and black circles mark the individual measurements. (f) Representative images of nodulation phenotypes of *GmNSP1a* or *GmNSP1b* knockout mutant hairy roots. Scale bars = 1 cm. (g) qRT-PCR analysis of *GmNIN1a* and *GmENOD40-1* expression in transgenic hairy roots carrying EV, *Cas9-GmNSP1a* and *Cas9-GmNSP1b*. Hairy roots were collected at 2 DAI. Expression levels were normalized to that of *GmELF1b*. Data are shown as means ± SD, and asterisks represent statistically significant differences relative to EV control. Two-sided Student's *t*-test, $*p < 0.05$; $**p < 0.01$; $***p < 0.001$.

a

	GmNSP1a sgRNA1 target<br>CTTTTGCTTCCAGCGAGCCGCGG	Editing Condition	*GmNSP1a* sgRNA2 target<br>CCTATACCGGACACGCAGAAGGG	Editing Condition
EV	CTTTTGCTTCCAGCGAGCCGCGG		CCTATACCGGACACGCAGAAGGG	
Cas9-GmNSP1a-1	CTTTTGCTTCCA - CGAGCCGCGG	-1bp	CC - - - ACCGGACACGCAGAAGGG	-3bp
Cas9-GmNSP1a-2	CTT - TGCTTCCAGCGAGCCGCGG	-1bp	CCTATACCGGTTACGCAGAAGGG	S-2bp
Cas9-GmNSP1a-3	CTTTTGCTTCCAGC - - - - CGCGG	-4bp	CCTATACCGGACACG - - GAAGGG	-2bp
Cas9-GmNSP1a-4	CTTTTGCTTCCAGCGAGCCGCGG	NO	CCTATACCGGACACGCA - AAGGG	-1bp
Cas9-GmNSP1a-5	CTTTTGCTT - - AGCGAGCCGCGG	-2bp	CC - - - - CCGGACACGCAGAAGGG	-4bp
Cas9-GmNSP1a-6	CTTTT - - TTCCAGCGAGCGGCGG	-2bp; S-1bp	CCTATACCGGA - - - GCAGAAGGG	-3bp
Cas9-GmNSP1a-7	CTTTTGCTTCCAG - - - - - CGCGG	-5bp	CCTATACCGGACACGCAGAAGGG	NO
Cas9-GmNSP1a-8	CT - - TGCTTCCAGCGAGCCGCGG	-2bp	CCTATACCGGAC - - GCAGAAGGG	-2bp
Cas9-GmNSP1a-9	CTTTTGCT - - - - - - GAGCCGCGG	-6bp	CCTAAACCGGACACGCACAAGGG	S-2bp
Cas9-GmNSP1a-10	CT - - - GCTTCCAGCGAGCCGCGG	-3bp	CCTATACCGGATACGCAGAAGGG	S-1bp
Cas9-GmNSP1a-11	CTTTTGCTTCCAGCGAGC - GCGG	-1bp	CCTATACCGGACAC - - AGAAGGG	-2bp
Cas9-GmNSP1a-12	CTTTTGCTTCCAGCGAGCCGCGG	NO	CCTATACC - GACACGCAGAAGGG	-1bp
Cas9-GmNSP1a-13	CTTTTGCTTCCAGCGAGCCGCGG	NO	CCTATACCGGACACGCAG - - GGG	-2bp
Cas9-GmNSP1a-14	CTTTTGCTTCCAGCG - - - - GCGG	-4bp	CCTATACCGGACAC - - AGAAGGG	-2bp
Cas9-GmNSP1a-15	CTTTTGGATCCAGCGAGCCGCGG	S-2bp	CC - - - ACCGGACACGCAGAAGGG	-3bp
Cas9-GmNSP1a-16	C - - TTGCTTCCAGCGAGCCGCGG	-2bp	CCTATACCGGA - ACGCAGAAGGG	-1bp
Cas9-GmNSP1a-17	CTTTTGCTTCCA - - - AGCCGCGG	-3bp	CCTATACCGGACACGCAGAAGGG	NO
Cas9-GmNSP1a-18	CT - TTACTTCCAGCG - - CCGCGG	-3bp; S-1bp	CCTATACCGGAC - CGCAGAAGGG	-1bp

b

	GmNSP1b sgRNA1 target<br>ACTCATGGAGAACATACAAGAGG	Editing Condition	*GmNSP1b* sgRNA2 target<br>GGTTCCTTCAAAACTCAGGCCGG	Editing Condition
EV	ACTCATGGAGAACATACAAGAGG		GGTTCCTTCAAAACTCAGGCCGG	
Cas9-GmNSP1b-1	ACTCATGGAG - - CATACAAGAGG	-2bp	GGTTCCTTC - - AACTCAGGCCGG	-2bp
Cas9-GmNSP1b-2	AC - - - - GGAGAACATACAAGAGG	-4bp	GGTTCCTTCAAAACTCAGGCCGG	NO
Cas9-GmNSP1b-3	ACTCATGG - - - - - - TACAAGAGG	-6bp	GGTTCCTTCAAAACTCACGCCGG	S-1bp
Cas9-GmNSP1b-4	ACTCATGGAGAACATACAAGAGG	NO	GGTTCCTTCAA - ACTCAGGCCGG	-1bp
Cas9-GmNSP1b-5	ACTCATGGAGAACATA - - - GAGG	-3bp	GG - - CCTTCAAAACTCAGGCCGG	-2bp
Cas9-GmNSP1b-6	ACTCATTGAGAACATACAAGAGG	S-1bp	GGTTCCTTC - AAACTCAGGCCGG	-1bp
Cas9-GmNSP1b-7	ACTCATGGAGA - - - - - CAAGAGG	-5bp	GGTTCCTTCAAAACTCAGGCCGG	NO
Cas9-GmNSP1b-8	ACTCATCTAGAACATACAAGAGG	S-2bp	GGTTCCTTCAA - - CTCAGGCCGG	-2bp
Cas9-GmNSP1b-9	AC - - - TGGAGAACATACAAGAGG	-3bp	GGTTCCTTCAAAACTC - GGCCGG	-1bp
Cas9-GmNSP1b-10	ACTCATGGAGAACATACCAAGAGG	+1bp	GGTTCCTTCA - - - CTCAGGCCGG	-3bp
Cas9-GmNSP1b-11	ACTCATGGAGAA - - - ACAAGAGG	-3bp	GGTTCCTTCAAAACTCAGGGCGG	S-1bp
Cas9-GmNSP1b-12	ACACATGGAG - ACATACAAGAGG	-1bp; S-1bp	GGTTCCTTCAAAACTCAGGCCGG	NO
Cas9-GmNSP1b-13	ACTCAT - - AGAACATACAAGAGG	-2bp	GGTTCC - TCAAAACTCAGGCCGG	-1bp
Cas9-GmNSP1b-14	ACTCATGGAGAACA - - - - - GAGG	-5bp	GGTTCCTTCAAAACTCCGGCCGG	S-1bp
Cas9-GmNSP1b-15	ACTCATGGAGA - CATACAAGAGG	-1bp	GGTTCCTTCAAA - CTCAGGCCGG	-1bp
Cas9-GmNSP1b-16	ACTCATCGAGAACATACAAGAGG	S-1bp	GG - - - CTTCAAAACTCAGGCCGG	-3bp
Cas9-GmNSP1b-17	ACTCATGGAGAACATACAAGAGG	NO	GGTTCCTTC - - AACTCAGGCCGG	-2bp

Supplementary Figure 2. Gene editing conditions of sgRNAs per *GmNSP1a* or *GmNSP1b* knockout hairy root.

(a-b) The gene editing conditions in the *GmNSP1a* knockout hairy roots (n =18) (a) and *GmNSP1b* knockout hairy roots (n=17) (b). Each line in the bars indicates different gene editing condition in individual hairy roots. The letter "S" stands for base substitution, and "NO" stands for no editing occurs.

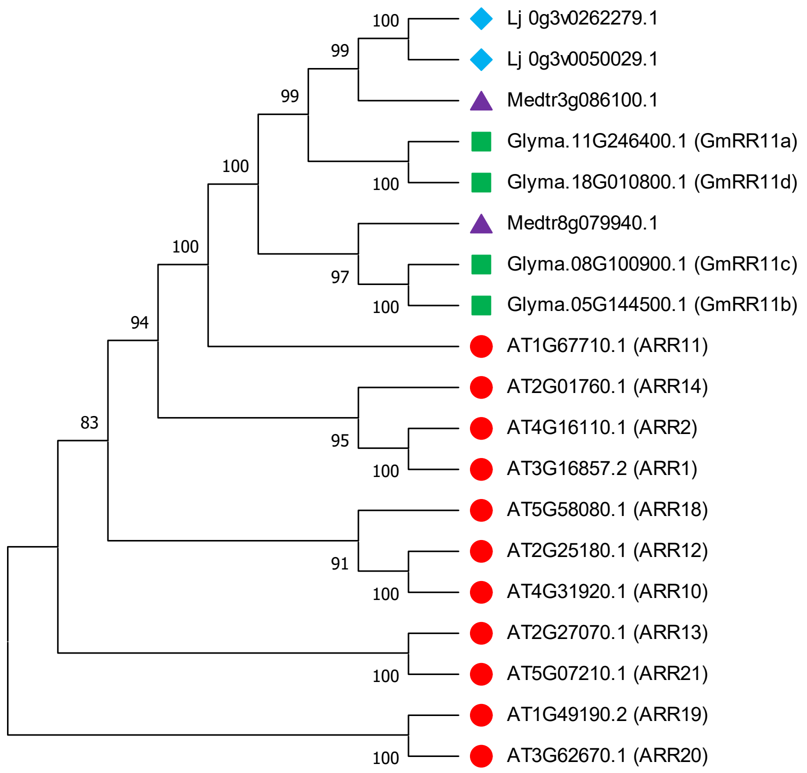

Supplementary Figure 3. Phylogenetic analysis of the RR11 homologs in *Arabidopsis thaliana*, *Louts japonicus*, *Medicago truncatula* and *Glycine max*.
The sequences of RR11 homologous proteins were obtained from Phytozome v12.1 database and the phylogenetic tree was built using MEGA7.0.

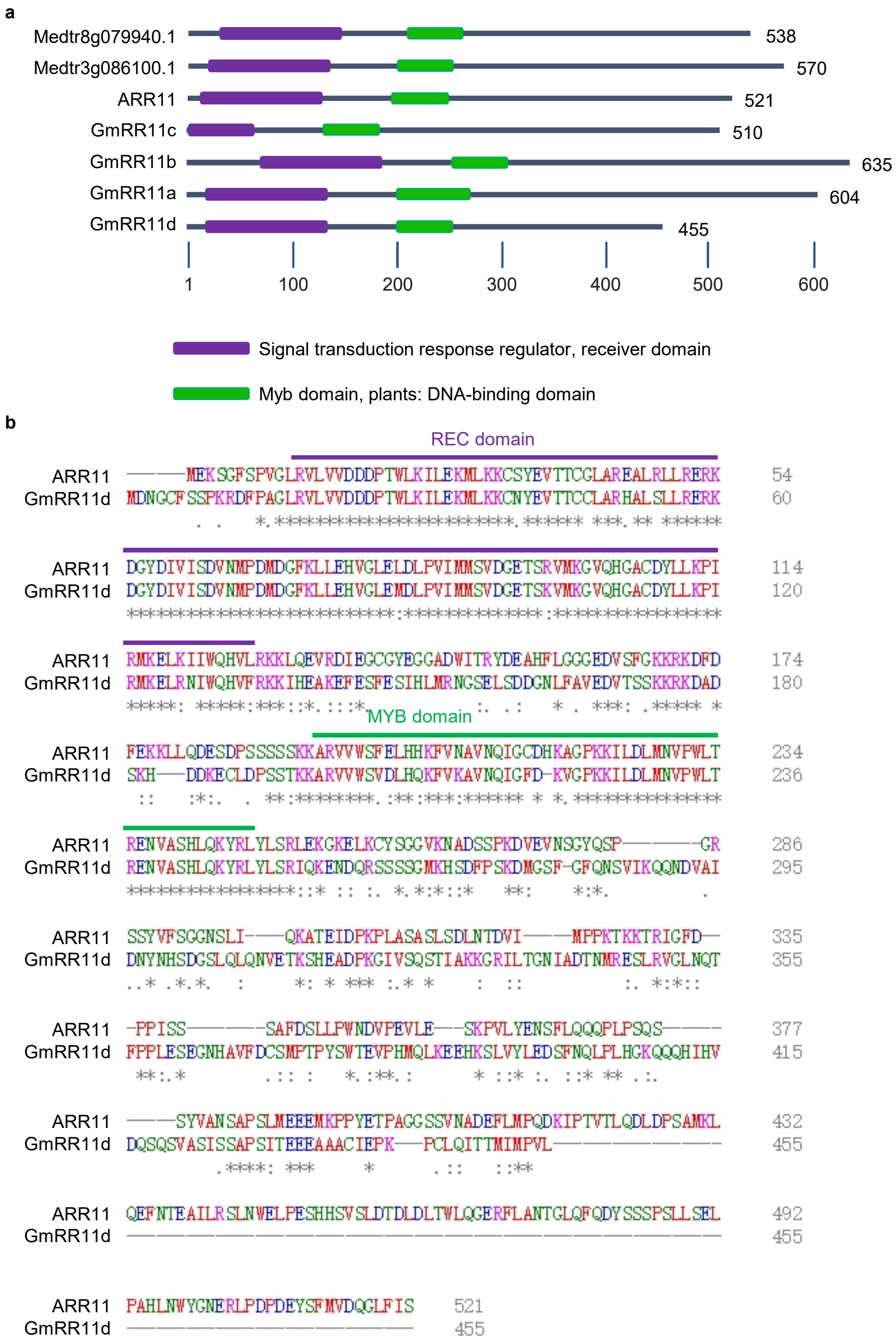

Supplementary Figure 4. Protein structure analysis and alignment of RR11 in different species.

(a) A schematic representation showing the protein structure of GmRR11d and its homologous proteins in *G. max*, *M. truncatula* and *A. thaliana*, which are represent B-type RRs conserved in REC and MYB domains but variable in C-terminal domains. (b) Amino acid sequences alignment of GmRR11d and ARR11. GmRR11d contains a receiver domain (namely REC domain, purple line) and GARP motif (namely MYB domain, green line), which has strong similarity with ARR11. The amino acid sequences of ARR11 and GmRR11d were submitted to Clustal Omega website (https://www.ebi.ac.uk/Tools/msa/clustalo/) for sequence alignment.

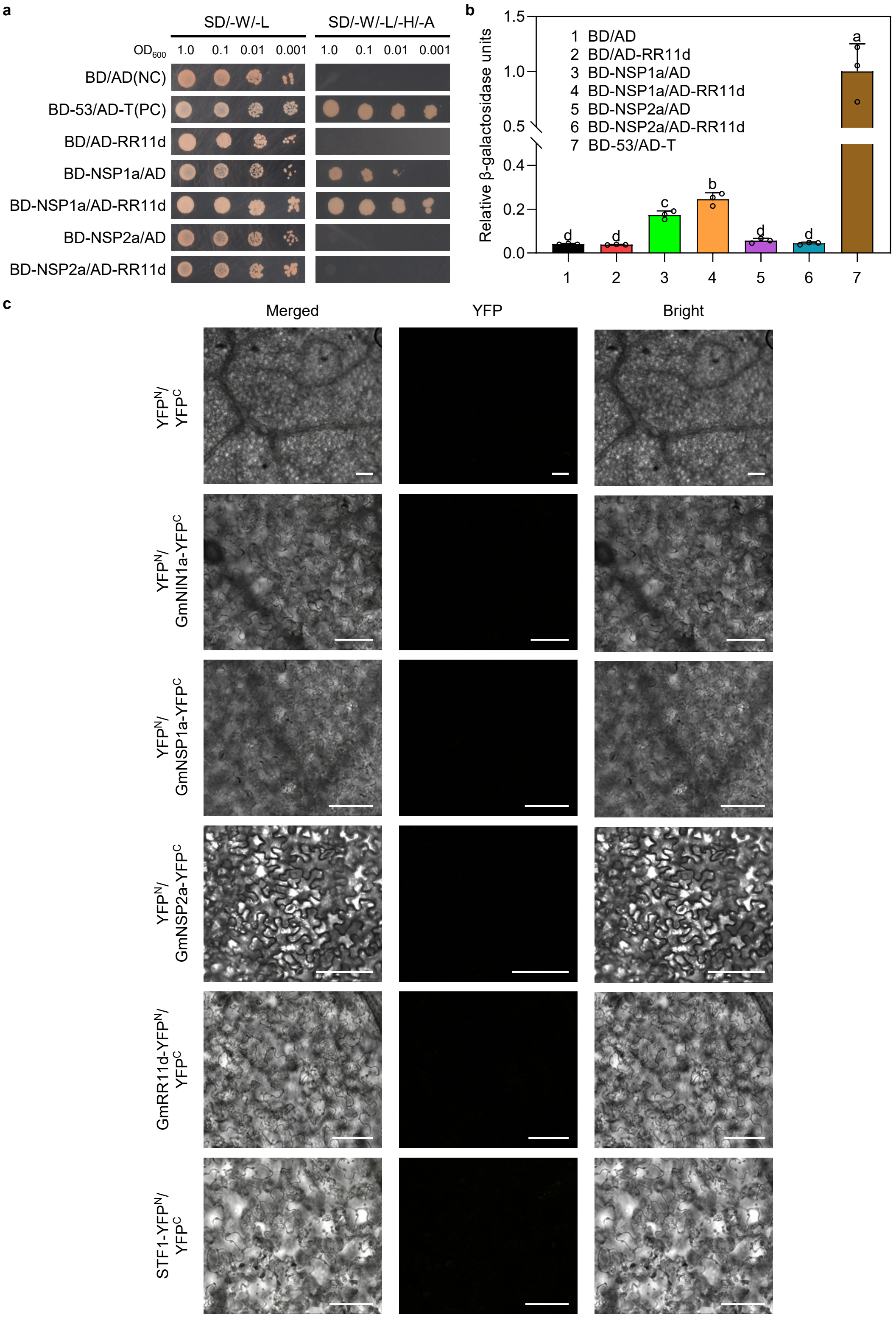

Supplementary Figure 5. GmRR11d interacts with GmNSP1a but not GmNSP2a in Y2H assay and negative controls of interaction assay between GmRR11d and GmNSP1a/2a in BiFC assay.

(a) Y2H analysis to detect the interactions between GmRR11d and GmNSP1a or GmNSP2a. Yeast cells co-transformed with pGADT7-GmRR11d/pGBKT7-GmNSP1a, pGADT7-GmRR11d/pGBKT7-GmNSP2a, pGADT7/pGBKT7-GmNSP1a,

pGADT7/pGBKT7-GmNSP2a, pGADT7-GmRR11d/pGBKT7 or pGADT7/pGBKT7 were cultured in selective media lacking Leu and Trp (SD/-2); and subsequently in media lacking Ade, His, Leu, and Trp (SD/-4) to test protein-protein interactions. (b) Quantification of β-galactosidase activity in *S. cerevisiae* revealed interactions between GmRR11d and GmNSP1a or GmNSP2a. Data are presented as means ± SD of three biological replicates. Different letters indicate significant differences at $p < 0.05$ (one-way ANOVA). (c) BiFC assay to detect the interaction between GmRR11d, GmNSP1a, GmNSP2a and empty control (YN or YC). GmNIN1a-YFP^C and STF1-YFP^N were used as controls. Scale bars = 100 μm. Four independent experiments were repeated with similar results.

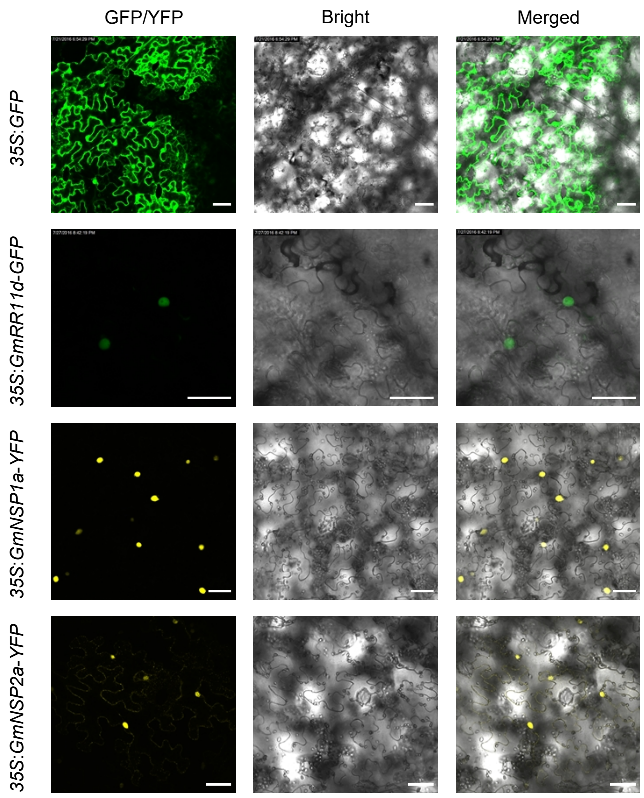

Supplementary Figure 6. Subcellular localization assays of GmRR11d-GFP, GmNSP1a-YFP and GmNSP2a-YFP in epidermal cells of tobacco leaves.

The constructs harboring *35S:GFP*, *35S:GmRR11d-GFP*, *35S:GmNSP1a-YFP* or *35S:GmNSP2a-YFP* were transformed into *N. benthamiana* leaves. The fluorescence of GFP or YFP in *N. benthamiana* leaf cells was observed at 36 hours after transformation. Scale bars = 50 μm. Three independent experiments were repeated with similar results.

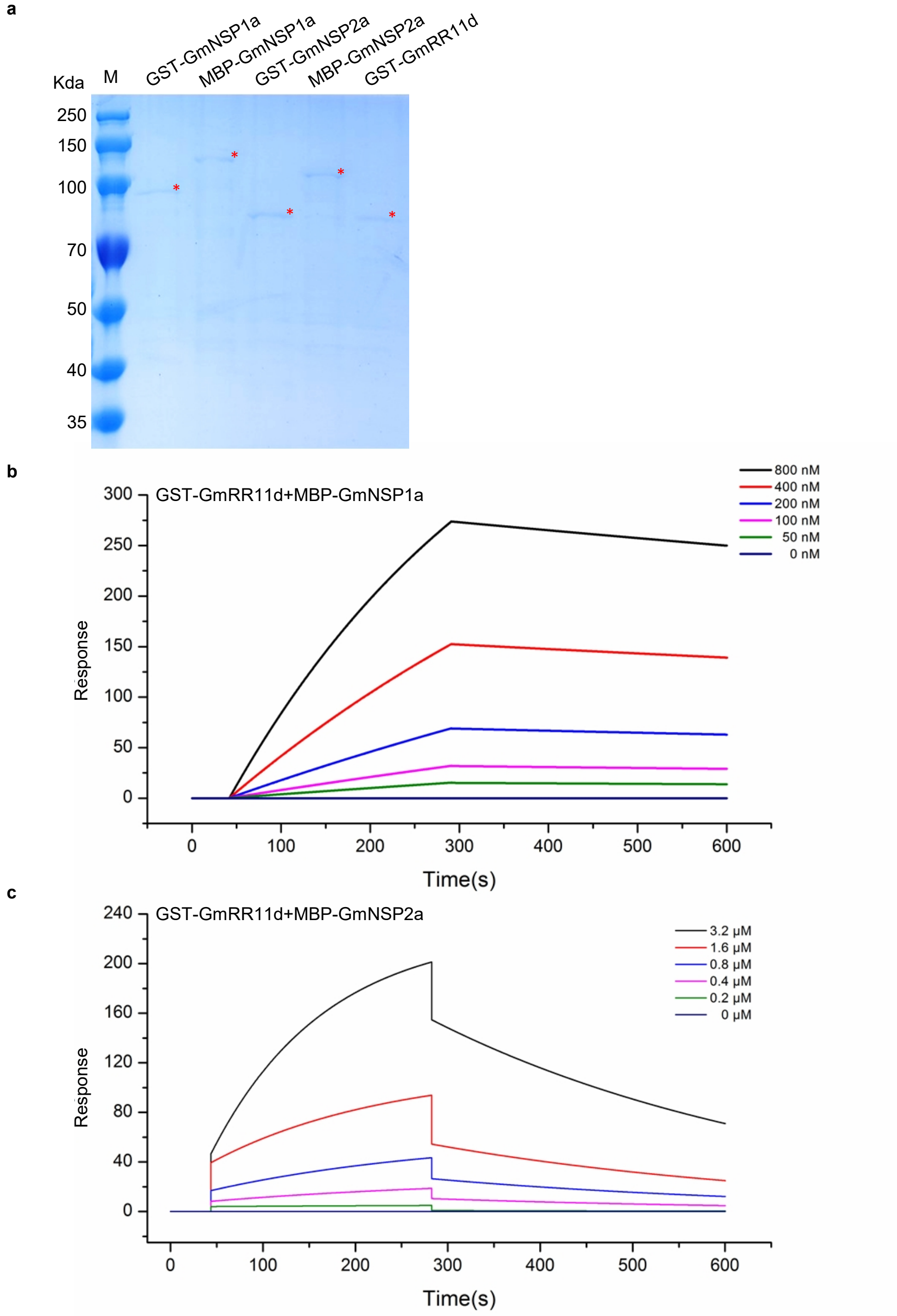

Supplementary Figure 7. LSPR assay for the interactions between GmRR11d and GmNSP1a/2a.

(a) The proteins of GST-GmNSP1a, MBP-GmNSP1a, GST-GmNSP2a, MBP-GmNSP2a and GST-GmRR11d were used in LSPR assay. Red stars indicate the specified proteins. Three independent experiments were repeated with similar results. (b) LSPR assay showing the interaction between MBP-GmNSP1a and GST-GmRR11d. (c) LSPR assay showing the weakly interaction between MBP-GmNSP2a and GST-GmRR11d. Lines with different colors in (b-c) are the concentrations of MBP-GmNSP1a (b) and MBP-GmNSP2a (c).

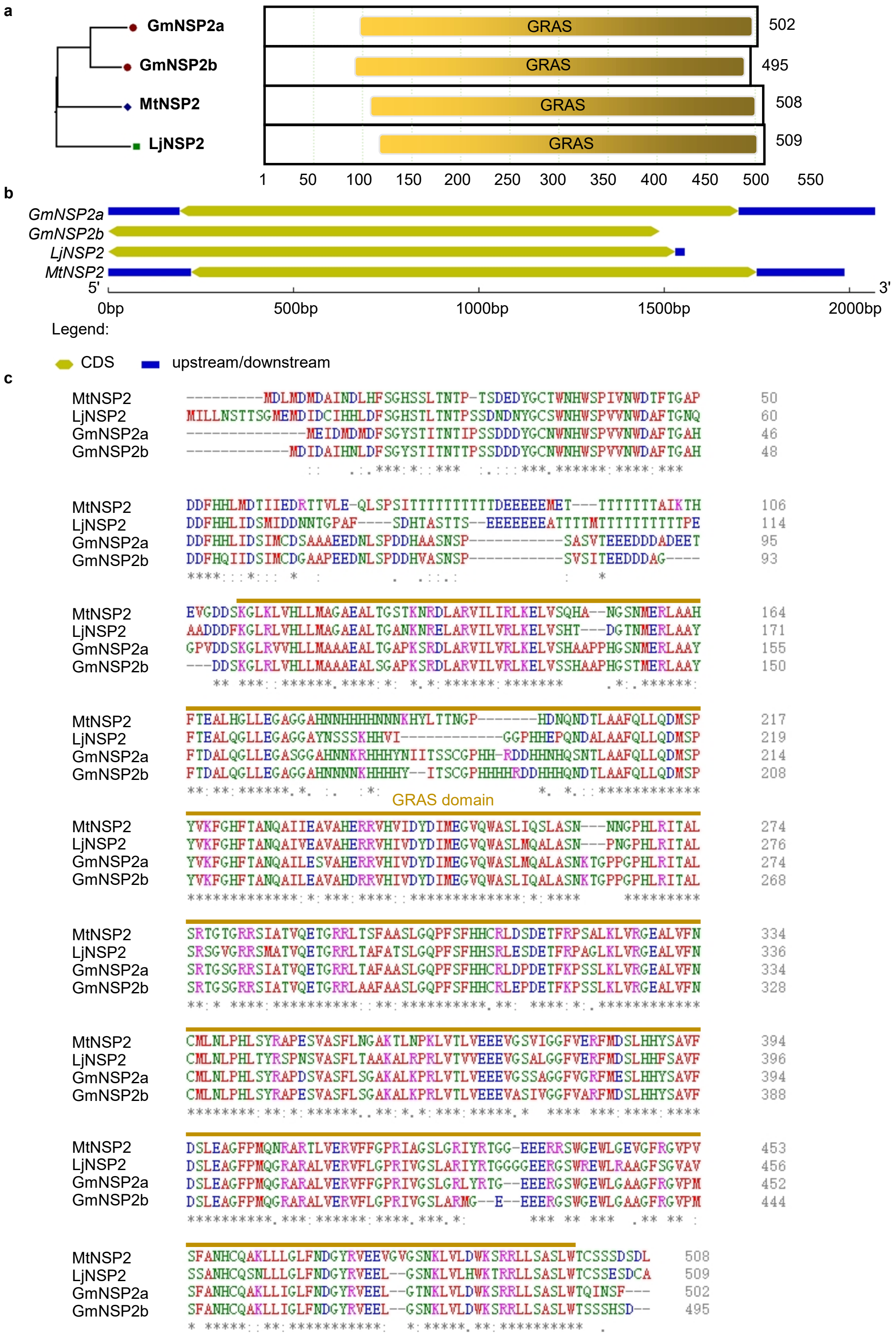

Supplementary Figure 8. Bioinformatics analysis of NSP2 proteins in legumes. (a) Phylogenetic relationships of the NSP2 proteins and their domain structures. (b) The gene structure analysis of *NSP2* in *Medicago truncatula*, *Lotus japonicus* and *Glycine max*. (c) Amino acid sequences of NSP2 proteins were aligned using Clustal Omega hosted by EMBL-EBI. NSP2s are highly conserved in *Medicago truncatula*, *Lotus japonicus* and *Glycine max*. The brown line indicates the GRAS domain.

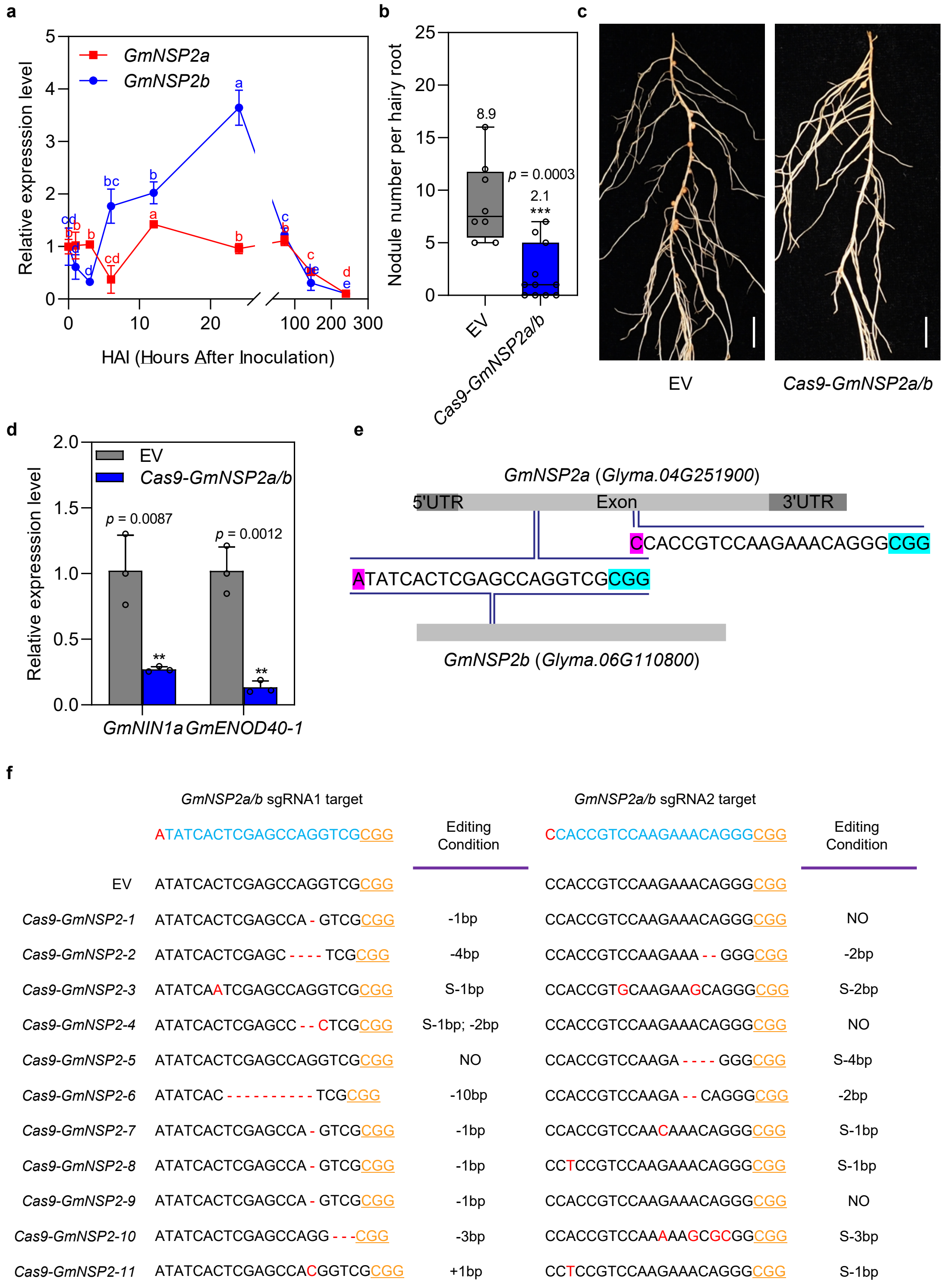

Supplementary Figure 9. Phenotype and expression analyses of *GmNSP2a/b* in double mutant hairy roots.

(a) The expression pattern of *GmNSP2a* and *GmNSP2b* by qRT-PCR. Seven-day-old seedlings were inoculated with *B. diazoefficiens* strain USDA110, and the roots at 0, 1, 3, 6, 12 HAI and 1, 3, 6, 10 DAI were used to test the expression of *GmNSP2a* and *GmNSP2b*. *GmELF1b* was used as the endogenous control gene. The data are shown as the means ± SD from three biological replicates. Different letters indicate significant

differences at $p < 0.05$ (One-way ANOVA). (b) Number of nodules per transgenic root expressing *Cas9-GmNSP2a/b*. More than 30 hairy roots were tested by sequencing and from which 8 hairy roots in EV and 11 hairy roots in *Cas9-GmNSP2a/b* were characterized as homozygous edited hairy roots and were calculated. Data are shown as means ± SD (n = 8 for EV and 11 for *Cas9-GmNSP2a/b*). Two-sided Student's *t*-test, $^{***}p < 0.001$. Boxes indicate the first and third quartiles and the whiskers indicate the minimum and maximum values, the black lines within the boxes indicate the median values and black circles mark the individual measurements. (c) Nodule number phenotypes of the transgenic hairy roots expressing *Cas9-GmNSP2a/b.* Scale bars = 1 cm. (d) The relative expression of *GmNIN1a*, *GmENOD40-1* in EV and *Cas9-GmNSP2a/b* transgenic roots at 2 DAI. Data are shown as means ± SD (n = 3 biological independent replicates). Asterisks indicate significant differences relative to the EV control. Two-sided Student's *t*-test, $^{**}p < 0.01$. (e) The diagram of two sgRNAs sequences of *GmNSP2a/b,* the sgRNAs that targeted both *GmNSP2a* and *GmNSP2b* were used. (f) The gene editing condition per *GmNSP2a/b* double mutant hairy roots identified (n = 11). The genes were edited in one sgRNA or both sgRNA sites. Each line in the bars indicates different gene editing condition in individual hairy roots. The letter "S" stands for base substitution and "NO" stands for no editing occurs.

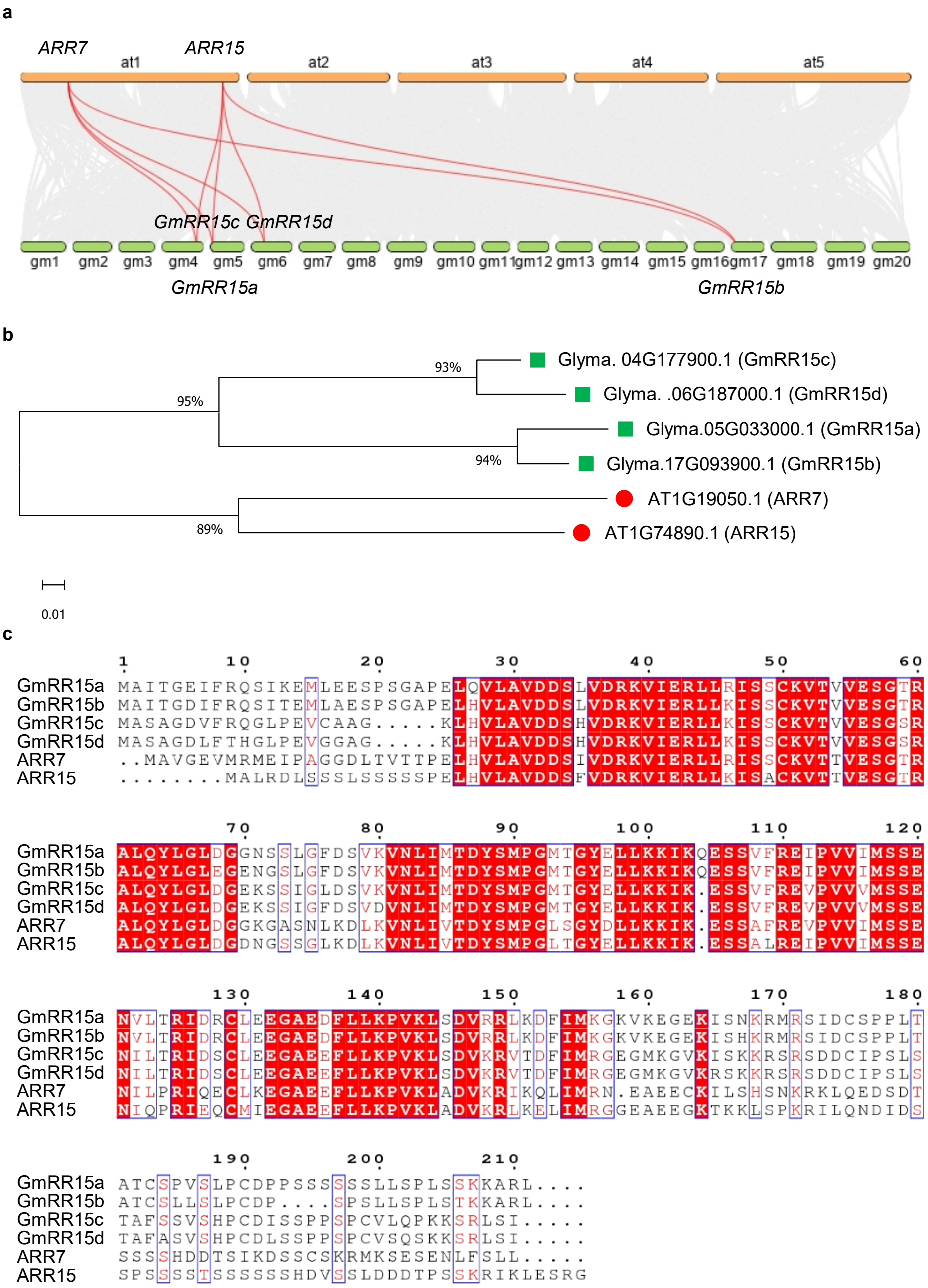

Supplementary Figure 10. Bioinformatics analysis of RR7 and RR15 in *Glycine max* and *A. thaliana*.

(a) Synteny plot analysis of ARR7 and ARR15 and its homologous protein in *Glycine max* (Gm). (b) Phylogenetic analysis of the ARR7 and ARR15 homologs in *Arabidopsis thaliana* and *Glycine max*. Four copies of ARR7 and ARR15 were named as GmRR15a, GmRR15b, GmRR15c and GmRR15d according to the homology with ARR7 and ARR15. (c) Amino acid sequences of GmRR15a/b/c/d, ARR7 and ARR15 were aligned using CLUSTALW website (https://www.genome.jp/tools-bin/clustalwand) and visualized by ESPript 3.0 website (http://espript.ibcp.fr/ESPript/cgi-bin/ESPript.cgi).

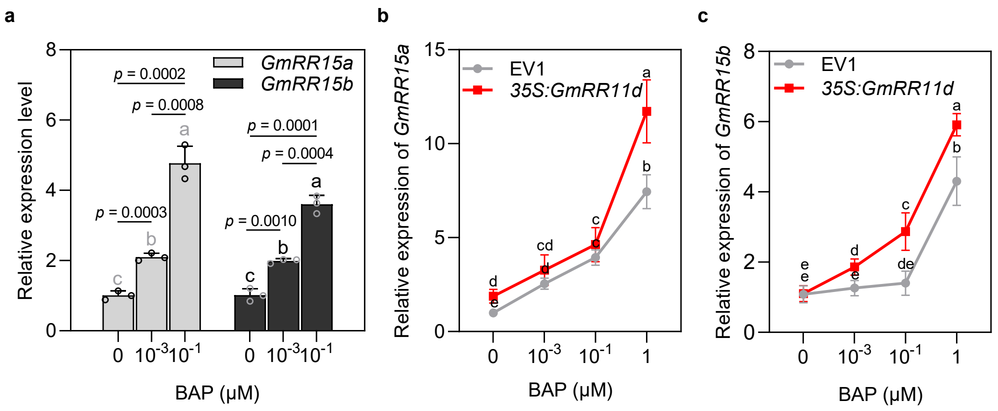

Supplementary Figure 11. GmRR11d induces *GmRR15a* and *GmRR15b* expression.

(a) Expression levels of *GmRR15a* and *GmRR15b* were analyzed in wild-type plants (W82) treated with different concentrations (0, 0.001, 0.1 μM) of BAP. Data are presented as means ± SD. More than 6 roots were analyzed in three independent biological repeats. Different letters indicate significant differences at $p < 0.05$ (One-way ANOVA). (b-c) Relative expression levels of cytokinin-responsive genes *GmRR15a* (b) and *GmRR15b* (c) from *35S*:*GmRR11d* transgenic hairy roots by qRT-PCR. The expression levels were normalized against the geometric mean of soybean *GmELF1b*. Data are presented as means ± SD. More than 6 roots were analyzed in three independent biological repeats. Different letters indicate significant differences at $p <$ 0.05 (Two-way ANOVA).

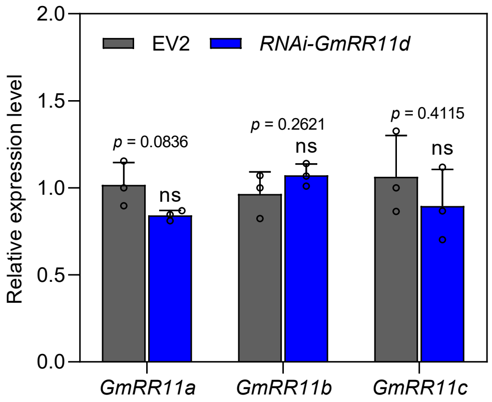

Supplementary Figure 12. The expression of *GmRR11a/b/c* in *RNAi-GmRR11d* transgenic roots.

qRT-PCR analysis of *GmRR11a/b/c* expression in empty vector (EV2) and *RNAi-GmRR11d* transgenic hairy roots. The expression levels were normalized against the geometric mean of soybean *GmELF1b*. Data are presented as means ± SD (n = 3 biological replicate). More than 12 roots were analyzed in three independent biological repeats. Two-sided Student's *t*-test was performed (ns: no significant difference).

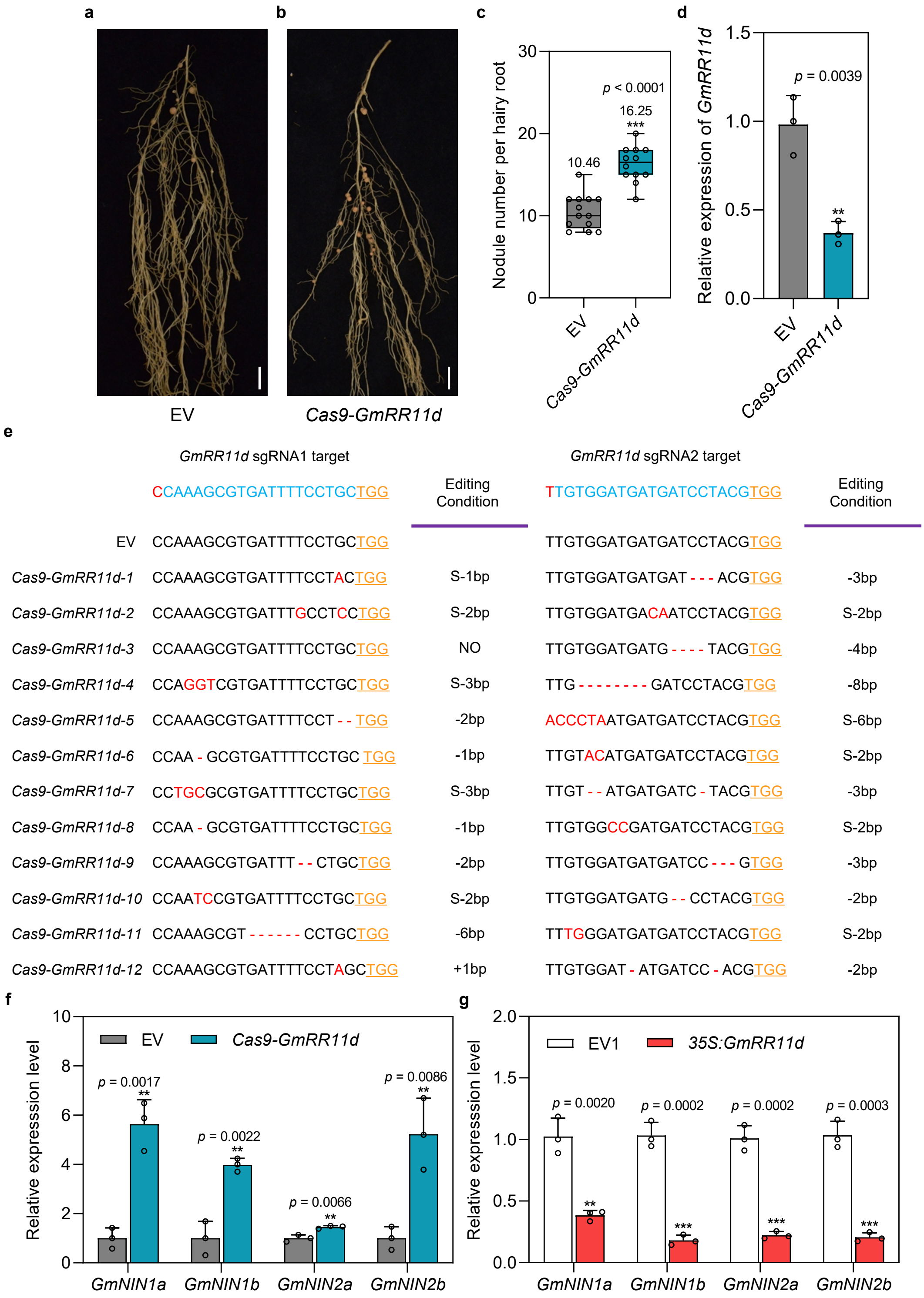

Supplementary Figure 13. Characterization of *Cas9-GmRR11d* hairy roots and analysis of *GmRR11d* on expression of *GmNINs* genes.

(a-b) Nodule number phenotypes of the transgenic hairy roots expressing EV (a) and *Cas9-GmRR11d* (b). Scale bars = 1 cm. (c) Number of nodules per transgenic root expressing EV and *Cas9-GmRR11d*. Data are presented as means ± SD (13 hairy roots in EV and 12 hairy roots in *Cas9-GmRR11d*). Asterisks indicate significant differences relative to the EV control. Two-sided Student's *t*-test, ****p* < 0.001. Boxes indicate the

first and third quartiles and the whiskers indicate the minimum and maximum values, the black lines within the boxes indicate the median values and black circles mark the individual measurements. (d) qRT-PCR analysis of *GmRR11d* expression in EV and *Cas9-GmRR11d* transgenic hairy roots. The expression levels were normalized against the geometric mean of soybean *GmELF1b*. Data are presented as means ± SD (n = 3 biological independent experiments). More than 12 roots were analyzed in three independent biological repeats. Asterisks indicate significant differences relative to the EV control. Two-sided Student's *t*-test, **$p < 0.01$. (e) The gene editing condition per *Cas9-GmRR11d* hairy roots identified. Each line in the bars indicates different gene editing condition in individual hairy roots. The letter "S" stands for base substitution and "NO" stands for no editing occurs. (f-g) The expression levels of *GmNIN1a/1b/2a/2b* in *Cas9-GmRR11d* (f) and *35S*:*GmRR11d* (g) transgenic hairy roots at 2 DAI. Data are presented as means ± SD (n = 3 biological independent experiments). Asterisks indicate significant differences relative to the empty control (EV or EV1). Two-sided Student's *t*-test, *$p < 0.05$; **$p < 0.01$; ***$p < 0.001$.

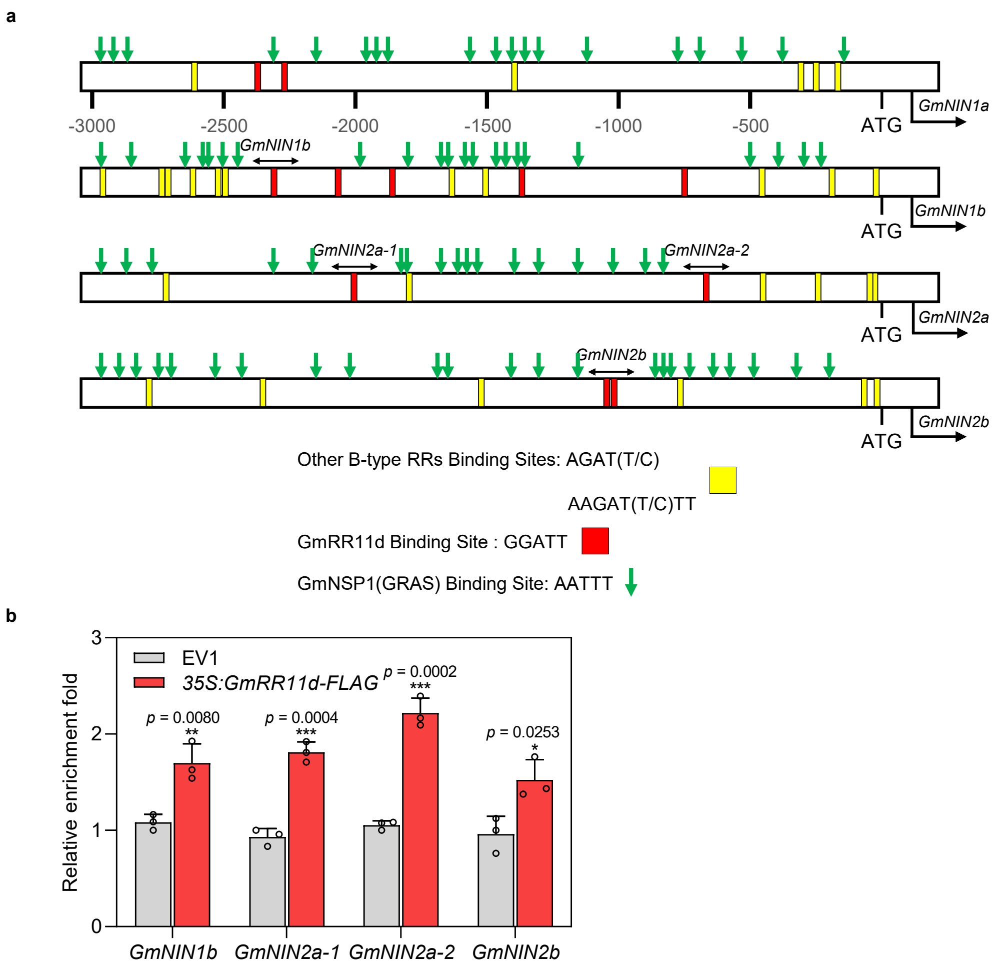

Supplementary Figure 14. Binding site analysis of the promoters of *GmNINs* and ChIP-qPCR analysis showing binding of GmRR11d to the *GmNIN* promoters.
(a) The promoter sequences (-3000 bp) upstream of four *GmNIN* genes were chosen to analyze the B-type RRs and GmNSP1 (GRAS domain) binding sites. The black double arrows represent the primers designed for ChIP-qPCR assays. (b) ChIP-qPCR assays showing binding of GmRR11d to *GmNIN* promoters. The DNA fragments were normalized to the input data. Data are presented as means ± SD of three biological repeats. Asterisks indicate significant differences relative to the EV1 control. Two-sided Student's *t*-test, *$p < 0.05$; **$p < 0.01$; ***$p < 0.001$.

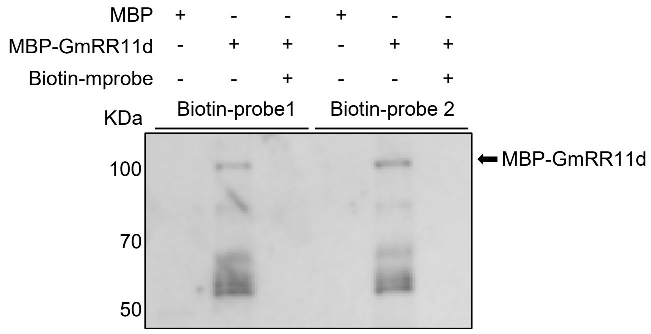

Supplementary Figure 15. GmRR11d protein interacts with *GmNIN1a* promoter fragment containing GGATT.

Protein-DNA pull-down assay showing GmRR11d protein interacts with oligo-DNAs containing GGATT in region A of *GmNIN1a* promoter. For the Biotin-mprobe, the GGATT sequence was changed to AAAAA. Three independent experiments were repeated with similar results.

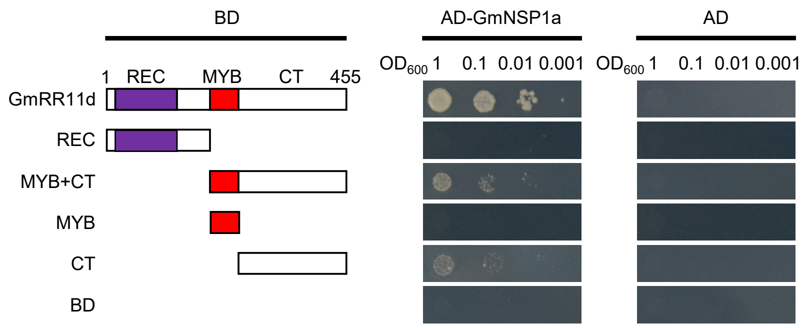

Supplementary Figure 16. Domain mapping for the interactions between GmRR11d or its derivatives and GmNSP1a by Y2H.

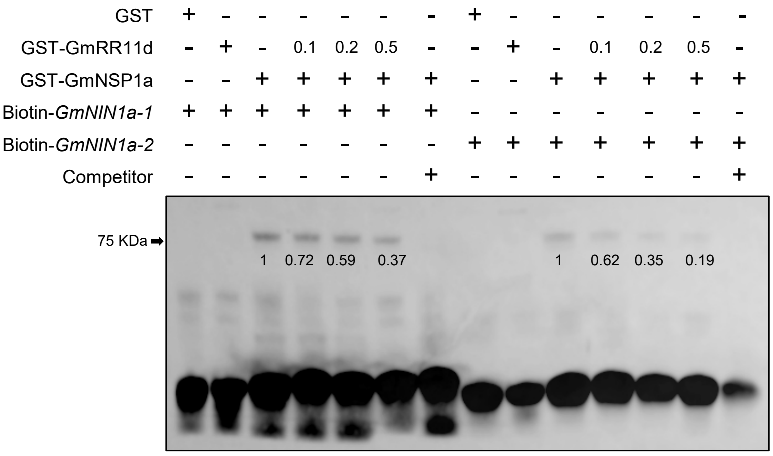

Supplementary Figure 17. GmRR11d negatively regulates the binding activity of GmNSP1a to the promoter of *GmNIN1a.*

0.2 μg GST-GmNSP1a was incubated with *GmNIN1a* promoter fragments containing GmNSP1a binding sites. For the competition of GmRR11d, an increasing amount of GST-GmRR11d (0.1 μg, 2 μg or 0.5 μg) was added in the reaction. The number below the band represents the relative gray value of the band, which is calculated by ImageJ software. Three independent experiments were repeated with similar results.

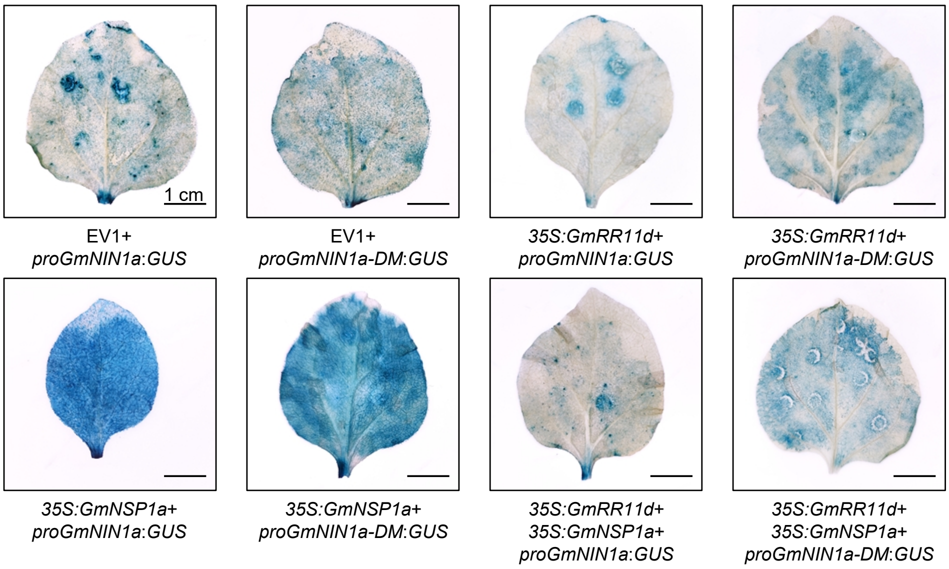

Supplementary Figure 18. GmRR11d inhibits GmNSP1a activated *GmNIN1a* expression.

The construct containing *proGmNIN1a*:*GUS* and *proGmNIN1a-DM*:*GUS* was transiently co-expressed with empty vector (EV1), *35S*:*GmRR11d*, *35S*:*GmNSP1a* or *35S*:*GmRR11d* + *35S*:*GmNSP1a* in the epidermal cells of *N. benthamiana* leaves. GUS expression was analyzed by GUS staining after 36 hours infection. Bars = 1 cm. Three independent experiments were repeated with similar results.

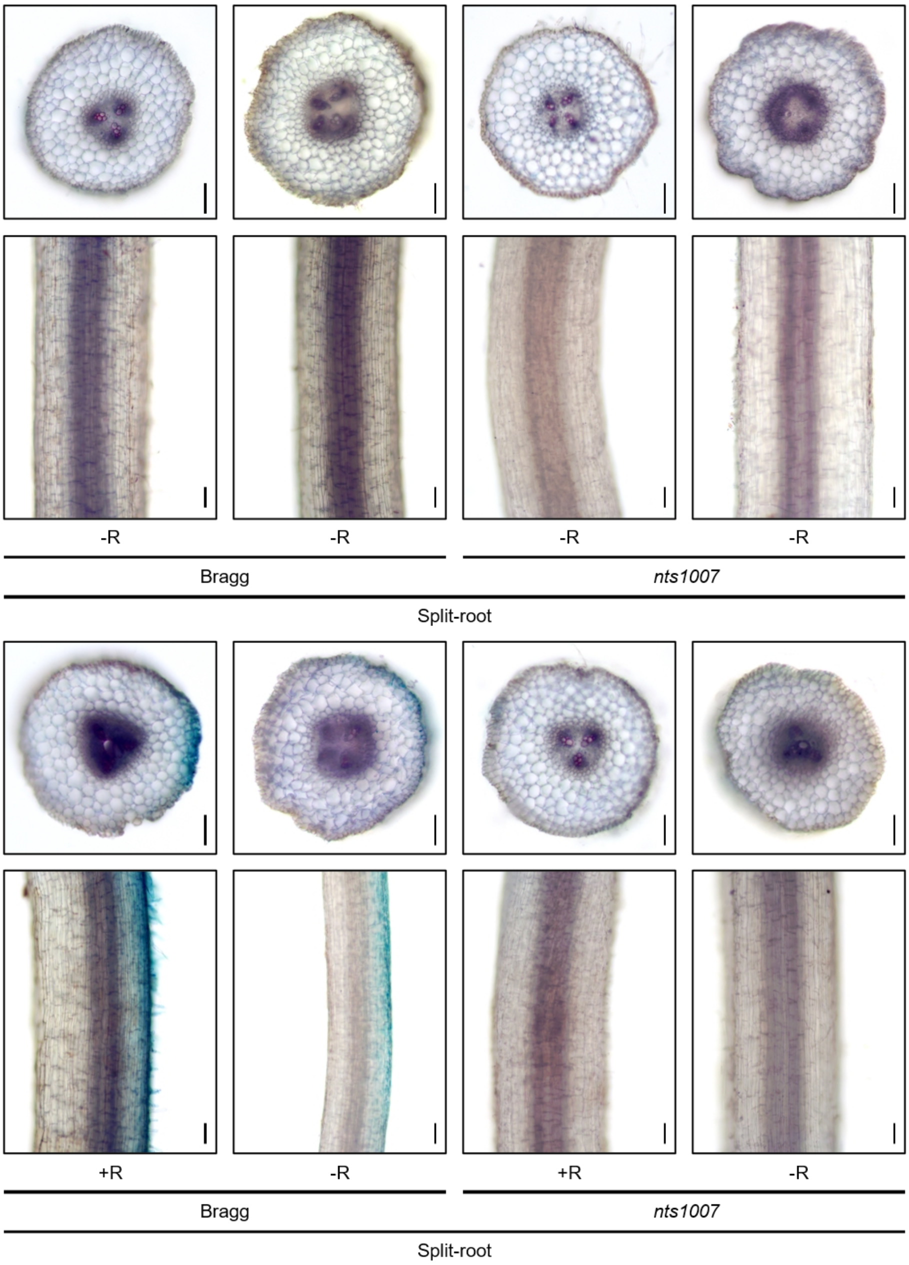

Supplementary Figure 19. *ProGmRR11d:GUS* assay in split-root system in Bragg and *nts1007* mutant.

ProGmRR11d:GUS was expressed in hairy roots of Bragg and *nts1007*, and the hairy roots from one seedling were split into two parts and one part was inoculated with *B. diazoefficiens* strain USDA110. The hairy roots were collected for GUS assay at 3 DAI. Bars = 100 μm. Three independent experiments were repeated with similar results.

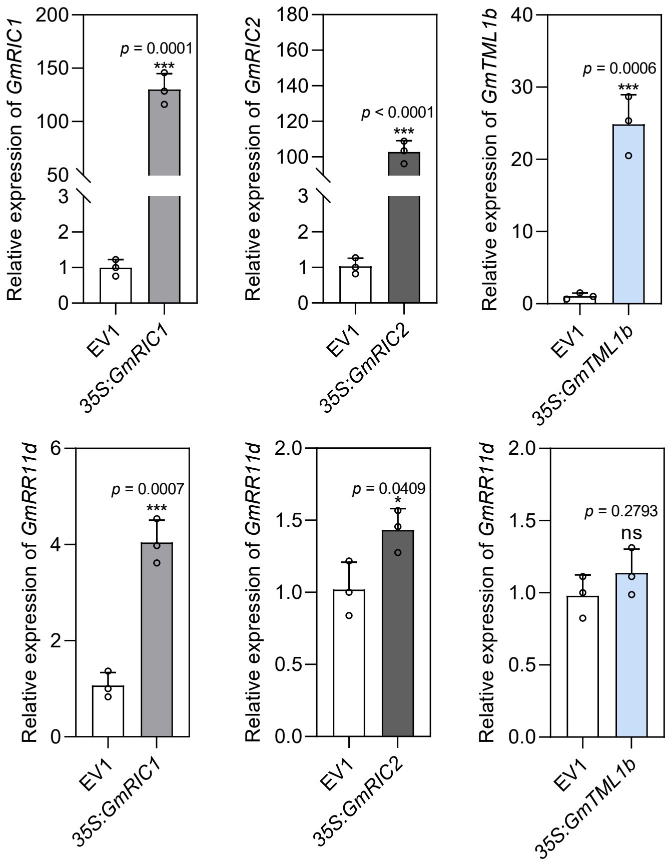

Supplementary Figure 20. The expression level of *GmRR11d* in *GmRIC1*, *GmRIC2* and *GmTML1b* overexpressing hairy roots.

The hairy roots overexpressing *GmRIC1*, *GmRIC2* or *GmTML1b* were collected at 3 DAI, and the expression of *GmRR11d* was analyzed by qRT-PCR. Data are presented as means ± SD from three biological repeats. More than 12 roots were analyzed in three independent biological repeats. Asterisks indicate significant differences relative to the EV1 control. Two-sided Student's *t*-test, $*p < 0.05$; $**p < 0.01$; $***p < 0.001$.

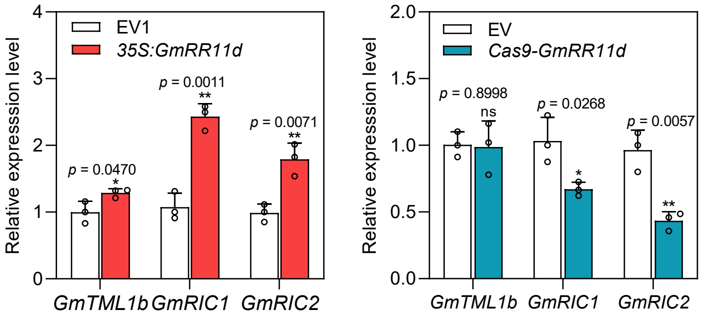

Supplementary Figure 21. The expression level of *GmRIC1*, *GmRIC2* and *GmTML1b* in *35S*:*GmRR11d* and *Cas9-GmRR11d* hairy roots.

The hairy roots overexpressing *GmRR11d* or *GmRR11d* mutant were collected at 3 DAI, and the expression of *GmRIC1*, *GmRIC2* and *GmTML1b* was analyzed by qRT-PCR. Data are presented as means ± SD from three biological repeats. More than 10 roots were analyzed in three independent biological repeats. Asterisks indicate significant differences relative to the empty vector control. Two-sided Student's *t*-test, $*p < 0.05$; $**p < 0.01$; $***p < 0.001$.

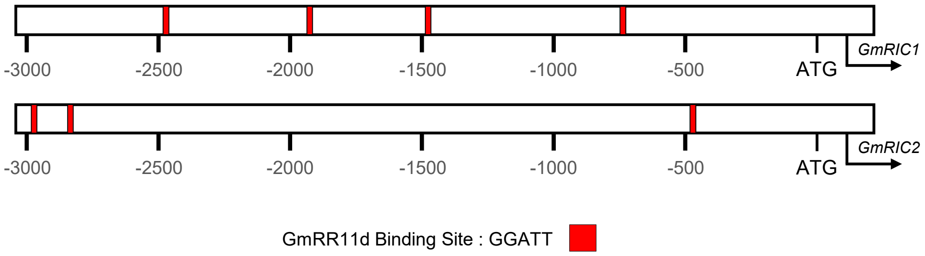

Supplementary Figure 22. Binding site analysis of GmRR11d on the promoters of *GmRIC1* and *GmRIC2*.

The promoter sequences (-3000 bp) upstream of *GmRIC1* and *GmRIC2* were chosen to analyze the GmRR11d binding sites.

Supplementary Table 1

The primers used in this study.

The primers for qRT-PCR, ChIP-qPCR and EMSA.

qRT-GmRR11d-F (Forward)	GTAACATTGCTGACACGAACAT
qRT-GmRR11d-R (Reverse)	GTTTCCTTCTGACTCTAGAGGG
qRT-GmRR11b-F	GTAAACAACCAGCAACAAAACG
qRT-GmRR11b-R	TTTGTGTCAGCAATGTTACCAG
qRT-GmRR11c-F	CTAAATGCACGAAAGGACGATT
qRT-GmRR11c-R	CTTCAACTCTTTGCTGAGAACC
qRT-GmRR11d-F	GTGTGATGACAAACGTTAAGCT
qRT-GmRR11d-R	GCTGAGAATCACTCTGGACTTA
qRT-GmRR15a-F	CCGACAGAGCATAAAGGAAATG
qRT-GmRR15a-R	CTATCAAACCCGAGAGAACTGT
qRT-GmRR15b-F	GCTATTACCGGCGATATTTTCC
qRT-GmRR15b-R	AAGAAATCTTCAGCAACCGTTC
qRT-GmELF1b-F	GTTGAAAAGCCAGGGGACA
qRT-GmELF1b-R	TCTTACCCCTTGAGCGTGG
qRT-GmNIN1a-F	TGGCGCACCATGCTAACAT
qRT-GmNIN1a-R	GGGTGTCATGGCAATCCTTT
qRT-GmENOD40-1-F	TCTCTCTTGAGTGGCAGAAGCA
qRT-GmENOD40-1-R	TGGAGTCCATTGCCTTTTCG
qRT-GmNSP1a-F	GATGCTAACCACAGGCTTGCA
qRT-GmNSP1a-R	GGCTCGCTGGAAGCAAAAG
qRT-GmNSP1b-F	CACTGAATGCTCAAAGTGTTGA
qRT-GmNSP1b-R	GTTCCTCAACACTGTCAGAAAC
qRT-GmNSP2a-F	TTATGGAGTCGCTGCATCACTATT
qRT-GmNSP2a-R	ATCCTTGGGCCGAAGAAAAC

qRT-GmNSP2b-F	AATCATTGCCAAGCGAAGCT
qRT-GmNSP2b-R	AGTCCAAAGCGAGGCAGAGA
ChIP-qRT-proGmNIN1a-F-A	CCACCCATCTAGCTAGCTTG
ChIP-qRT-proGmNIN1a-R-A	GTTGGGAGAAAGGGCATAG
ChIP-qRT-proGmNIN1a-F-B	TTCAGCTGTAGCTTCTTCGTAAT
ChIP-qRT-proGmNIN1a-R-B	TTAGCTTCTTTTTCATGCAAA
ChIP-qRT-proGmNIN1a-F-C	TTTGCATGAAAAAGAAGCTAA
ChIP-qRT-proGmNIN1a-R-C	AACTAACACGTAATAGGTAAATAAGCC
ChIP-qRT-proGmNIN1a-F-D	GGCTTATTTACCTATTACGTGTTAGTT
ChIP-qRT-proGmNIN1a-R-D	ATTGGAATGCACAACAAATGT
ChIP-qRT-proGmNIN1a-F-E	TCATATGTCTTTAGAGTACACTGATTAGC
ChIP-qRT-proGmNIN1a-R-E	TTAGATATAATAGGTCGACTTATTTATGTACAT
ChIP-qRT-proGmNIN1a-F-F	TTCAACTAACTATCTTAAAGTACGCAA
ChIP-qRT-proGmNIN1a-R-F	ATGAACAAACGAAATGTCTGAA
ChIP-qRT-proGmNIN1a-F-G	TTACATAGATACGAGCTAGCAGCTAG
ChIP-qRT-proGmNIN1a-	TTACGTACAGCACTTCGGC

R-G	
EMSA-GmRR11d-probe-1-bio-F	CGTGGTATCAACTTGGGATTAGGATGACAGAGTGC
EMSA-GmRR11d-probe-1-bio-R	GCACTCTGTCATCCTAATCCCAAGTTGATACCACG
EMSA-GmRR11d-probe-1-F	CGTGGTATCAACTTGGGATTAGGATGACAGAGTGC
EMSA-GmRR11d-probe-1-R	GCACTCTGTCATCCTAATCCCAAGTTGATACCACG
EMSA-GmRR11d-probe-2-bio-F	TTCAAAAAATCTGTAGGATTGCCACGTGGAACATG
EMSA-GmRR11d-probe-2-bio-R	CATGTTCCACGTGGCAATCCTACAGATTTTTTGAA
EMSA-GmRR11d-probe-2-F	TTCAAAAAATCTGTAGGATTGCCACGTGGAACATG
EMSA-GmRR11d-probe-2-R	CATGTTCCACGTGGCAATCCTACAGATTTTTTGAA
EMSA-GmNSP1a-probe-1-bio-F	TATAATTACAATTTTTCAAAAGCTATAATTTTAAAATAAC
EMSA-GmNSP1a-probe-1-bio-R	GTTATTTTAAAATTATAGCTTTTGAAAAATTGTAATTATA
EMSA-GmNSP1a-probe-1-F	TATAATTACAATTTTTCAAAAGCTATAATTTTAAAATAAC
EMSA-GmNSP1a-probe-1-R	GTTATTTTAAAATTATAGCTTTTGAAAAATTGTAATTATA
EMSA-GmNSP1a-probe-2-bio-F	GTGTTAGTTTTTAAATTTGATTTGAACTTTTTA
EMSA-GmNSP1a-probe-2-bio-R	TAAAAAGTTCAAATCAAATTTAAAAAACTAACAC

EMSA-GmNSP1a-probe-2-F	GTGTTAGTTTTTAAATTTGATTTGAACTTTTTA
EMSA-GmNSP1a-probe-2-R	TAAAAAGTTCAAATCAAATTTAAAAACTAACAC

The primers for plasmid construction.

Cas9-GmNSP1a-DT1-BsF	ATATATGGTCTCGATTGTTTTGCTTCCAGCGAGCCGGTT
Cas9-GmNSP1a-DT2-BsR	ATTATTGGTCTCGAAACTTCTGCGTGTCCGGTATAGCAA
Cas9-GmNSP1a-DT1-F0	TGTTTTGCTTCCAGCGAGCCGGTTTTAGAGCTAGAAATAGC
Cas9-GmNSP1a-DT2-R0	AACTTCTGCGTGTCCGGTATAGCAATCTCTTAGTCGACTCTAC
Cas9-GmNSP1b-DT1-BsF	ATATATGGTCTCGATTGCTCATGGAGAACATACAAGGTT
Cas9-GmNSP1b-DT2-BsR	ATTATTGGTCTCGAAACGCCTGAGTTTTGAAGGAACCAA
Cas9-GmNSP1b-DT1-F0	TGCTCATGGAGAACATACAAGGTTTTAGAGCTAGAAATAGC
Cas9-GmNSP1b-DT2-R0	AACGCCTGAGTTTTGAAGGAACCAATCTCTTAGTCGACTCTAC
Cas9-GmNSP1a/b-DT1-BsF	ATATATGGTCTCGATTGCTCATGGAGAACATACGAGGTT
Cas9-GmNSP1a/b -DT2-BsR	ATTATTGGTCTCGAAACCGGTTCTTCCAAAAGACACCAA
Cas9-GmNSP1a/b -DT1-F0	TGCTCATGGAGAACATACGAGGTTTTAGAGCTAGAAATAGC
Cas9-GmNSP1a/b-DT2-	AACCGGTTCTTCCAAAAGACACCAATCTCTTAGT

R0	CGACTCTAC
Cas9-GmNSP2a/b-DT1-BsF	ATATATGGTCTCGATTGTATCACTCGAGCCAGGTCGGTT
Cas9-GmNSP2a/b -DT2-BsR	ATTATTGGTCTCGAAACCCCTGTTTCTTGGACGGTGCAA
Cas9-GmNSP2a/b -DT1-F0	TGTATCACTCGAGCCAGGTCGGTTTTAGAGCTAGAAATAGC
Cas9-GmNSP2a/b -DT2-R0	AACCCCTGTTTCTTGGACGGTGCAATCTCTTAGTCGACTCTAC
35S:GmRR11d-FLAG-F /YN-GmRR11d-F	GGGGACAAGTTTGTACAAAAAAGCAGGCTTCATGGATAATGGTTGTTTCTCT
35S:GmRR11d-FLAG-R /YN-GmRR11d-R	GGGGACCACTTTGTACAAGAAAGCTGGGTCCAGAACTGGCATAATCATTG
35S:GmNSP1a-GFP-F /YC-GmNSP1a-F	GGGGACAAGTTTGTACAAAAAAGCAGGCTTCATGATCATGGAACCAAATCC
35S:GmNSP1a-GFP-R /YC-GmNSP1a-R	GGGGACCACTTTGTACAAGAAAGCTGGGTCTGATGTAAATGTTGAGGTCTTATAATTC
35S:GmNSP2a-GFP-F /YC-GmNSP2a-F	GGGGACAAGTTTGTACAAAAAAGCAGGCTTCATGGAAATAGACATGGACATG
35S:GmNSP2a-GFP-R /YC-GmNSP2a-F	GGGGACCACTTTGTACAAGAAAGCTGGGTCTTAAAATGAATTAATCTGAGTCC
RNAi-GmRR11d-F	GGGGACAAGTTTGTACAAAAAAGCAGGCTTCATGGATAATGGTTGTTTCTCT
RNAi-GmRR11d-R	GGGGACCACTTTGTACAAGAAAGCTGGGTCCAGAACTGGCATAATCATTG
proGmNIN1a-F	TAGCTCAAGTGAAAGCGATGTCTATG
proGmNIN1a-R	CTGCACCAACCCACCATATTCC
proGmRR11d-F	GGGGACAAGTTTGTACAAAAAAGCAGGCTTCGTAGTACTAGTCCAGTTCAATAGGGAAG

proGmRR11d-R	GGGGACCACTTTGTACAAGAAAGCTGGGTCTTTT GTGAGAGATACAGAAA AGAGG
BAR-F	AAGGATAGTGGGATTGTGCG
BAR-R	AGTCGGGAAACCTGTCGTG
GFP-F	ATGGTGAGCAAGGGCGAG
GFP-R	GCTCGTCCATGCCGAGAG

The primers for Y2H.

Y2H-GW-GmNSP1a-NT-F	GGGGACAAGTTTGTACAAAAAAGCAGGCTTCATG ATCATGGAACCAAATCCA
Y2H-GW-GmNSP1a-NT-R	GGGGACCACTTTGTACAAGAAAGCTGGGTCATTT CCATTGGCCTTAGCT
Y2H-GW-GmNSP1a-LHR1-F	GGGGACAAGTTTGTACAAAAAAGCAGGCTTCATG AACTGCAACAACAAGGATGG
Y2H-GW-GmNSP1a-LHR1-R	GGGGACCACTTTGTACAAGAAAGCTGGGTCAGA GGGAGAAGATGATAGATGTT
Y2H-GW-GmNSP1a-F	GGGGACAAGTTTGTACAAAAAAGCAGGCTTCATG ATCATGGAACCAAATCCA
Y2H-GW-GmNSP1a-R	GGGGACCACTTTGTACAAGAAAGCTGGGTCTGAT GTAAATGTTGAGGTCTTATAATTC
Y2H-GW-GmNSP1a-VHIID-F	GGGGACAAGTTTGTACAAAAAAGCAGGCTTCATG TCAGGGTCTATAACTTTTGCT
Y2H-GW-GmNSP1a-VHIID-R	GGGGACCACTTTGTACAAGAAAGCTGGGTCTGTG TCATTTTCAGTGGAGG
Y2H-GW-GmNSP1a-LHR2-F	GGGGACAAGTTTGTACAAAAAAGCAGGCTTCATG CCATTTTGTATTGGTCCT
Y2H-GW-GmNSP1a-LHR2-R	GGGGACCACTTTGTACAAGAAAGCTGGGTCATTC AACTGATGCAACCTAA
Y2H-GW-GmNSP1a-	GGGGACAAGTTTGTACAAAAAAGCAGGCTTCATG

PFYRE-F	CACAATGCCCCTGATGAA
Y2H-GW-GmNSP1a-PFYRE-R	GGGGACCACTTTGTACAAGAAAGCTGGGTCCGCAGCCTCACCTTC
Y2H-GW-GmNSP1a-SAW-F	GGGGACAAGTTTGTACAAAAAAGCAGGCTTCATGAAGGCATTGACAAACCAG
Y2H-GW-GmNSP1a-SAW-R	GGGGACCACTTTGTACAAGAAAGCTGGGTCTGATGTAAATGTTGAGGTCTTATAATTC
Y2H-GW-GmRR11d-F	GGGGACAAGTTTGTACAAAAAAGCAGGCTTCATGGATAATGGTTGTTTCTCTTC
Y2H-GW-GmRR11d-R	GGGGACCACTTTGTACAAGAAAGCTGGGTCCAGAACTGGCATAATCATTGTT
Y2H-GW-GmRR11d-CT-F	GGGGACAAGTTTGTACAAAAAAGCAGGCTTCATGTATTTGAGTAGGATT
Y2H-GW-GmRR11d-CT-R	GGGGACCACTTTGTACAAGAAAGCTGGGTCCAGAACTGGCATAATCATTGTT
Y2H-GW-GmRR11d-REC-F	GGGGACAAGTTTGTACAAAAAAGCAGGCTTCATGGATAATGGTTGTTTCTCTTC
Y2H-GW-GmRR11d-REC-R	GGGGACCACTTTGTACAAGAAAGCTGGGTCCTTGGTGGATGATGG
Y2H-GW-GmRR11d-MYB-F	GGGGACAAGTTTGTACAAAAAAGCAGGCTTCATGCCATCATCCACCAAG
Y2H-GW-GmRR11d-MYB-R	GGGGACCACTTTGTACAAGAAAGCTGGGTCAAGCCTGTATTTCTGCAAGTG
Y2H-GW-GmNSP1b-F	GGGGACAAGTTTGTACAAAAAAGCAGGCTTCATGATCATGGAACCAAACCCAA
Y2H-GW-GmNSP1b-R	GGGGACCACTTTGTACAAGAAAGCTGGGTCTGTTGAGCTCTGATCATTCCC
Y2H-GW-GmNSP2a-F	GGGGACAAGTTTGTACAAAAAAGCAGGCTTCATGGAAATAGACATGGACATGG

Y2H-GW-GmNSP2a-R	GGGGACCACTTTGTACAAGAAAGCTGGGTCAAATGAATTAATCTGAGTCCAAAG
Y2H-GW-GmNSP2b-F	GGGGACAAGTTTGTACAAAAAAGCAGGCTTCATGGACATAGATGCTATCCACAA
Y2H-GW-GmNSP2b-R	GGGGACCACTTTGTACAAGAAAGCTGGGTCTCTGAGTGGGAGGAAGAAGTC

The primers for pull-down assay.

MBP-GmNSP2a-EcoRI-F	CCGGAATTCATGGAAATAGACATGGACATG
MBP-GmNSP2a-BamHI-R	CGGGATTCTTAAAATGAATTAATCTGAGTCC
GST-GmNSP2a-BamHI-F	CGGGATTCATGGAAATAGACATGGACATG
GST-GmNSP2a-EcoRI-R	CCGGAATTCAAATGAATTAATCTGAGTCC
MBP/GST-GmRR11d-EcoRI-F	GGAATTCCCAACAAACAAACCCAACCT
MBP/GST-GmRR11d-SalI-R	GCGTCGACCAGAACTGGCATAATCATTGTTG
MBP/GST-GmNSP1a-EcoRI-F	GGAATTCATGATCATGGAACCAAATCC
MBP/GST-GmNSP1a-SalI-R	GCGTCGACTGATGTAAATGTTGAGGTCTTATAATTC
